# Comparative transcriptome analysis of coleorhiza development in *japonica* and *Indica* rice

**DOI:** 10.1186/s12870-021-03276-z

**Published:** 2021-11-04

**Authors:** Tao Song, Debatosh Das, Neng-Hui Ye, Guan-Qun Wang, Fu-Yuan Zhu, Mo-Xian Chen, Feng Yang, Jian-Hua Zhang

**Affiliations:** 1 Co-Innovation Center for Sustainable Forestry in Southern China, College of Biology and the Environment, Nanjing Forestry University, Nanjing, 210037 China; 2 Shenzhen Research Institute, The Chinese University of Hong Kong, Shenzhen, 518057 China; 3 Southern Regional Collaborative Innovation Center for Grain and Oil Crops in China, College of Agriculture, Hunan Agricultural University, Changsha, 410128 China; 4 Department of Biology, Hong Kong Baptist University, Kowloon, Hong Kong; 5 CAS Key Laboratory of Quantitative Engineering Biology, Shenzhen Institute of Synthetic Biology, Shenzhen Institute of Advanced Technology, Chinese Academy of Sciences, Shenzhen, 518055 China; 6 School of Life Sciences and State Key Laboratory of Agrobiotechnology, The Chinese University of Hong Kong, Shatin, Hong Kong

**Keywords:** Transcriptomics, Abscisic acid, Auxin, Coleorhiza hair, *Japonica*, *Indica*, Water

## Abstract

**Background:**

Coleorhiza hairs, are sheath-like outgrowth organs in the seeds of *Poaceae* family that look like root hair but develop from the coleorhiza epidermal cells during seed imbibition. The major role of coleorhiza hair in seed germination involves facilitating water uptake and nutrient supply for seed germination. However, molecular basis of coleorhiza hair development and underlying genes and metabolic pathways during seed germination are largely unknown and need to be established.

**Results:**

In this study, a comparative transcriptome analysis of coleorhiza hairs from *japonica* and *indica* rice suggested that DEGs in embryo samples from seeds with embryo in air (EIA) as compared to embryo from seeds completely covered by water (CBW) were enriched in water deprivation, abscisic acid (ABA) and auxin metabolism, carbohydrate catabolism and phosphorus metabolism in coleorhiza hairs in both cultivars. Up-regulation of key metabolic genes in ABA, auxin and dehydrin and aquaporin genes may help maintain the basic development of coleorhiza hair in *japonica* and *indica* in EIA samples during both early and late stages. Additionally, DEGs involved in glutathione metabolism and carbon metabolism are upregulated while DEGs involved in amino acid and nucleotide sugar metabolism are downregulated in EIA suggesting induction of oxidative stress-alleviating genes and less priority to primary metabolism.

**Conclusions:**

Taken together, results in this study could provide novel aspects about the molecular signaling that could be involved in coleorhiza hair development in different types of rice cultivars during seed germination and may give some hints for breeders to improve seed germination efficiency under moderate drought conditions.

**Supplementary Information:**

The online version contains supplementary material available at 10.1186/s12870-021-03276-z.

## Background

Coleorhiza hair can form under dry soil conditions and can’t be induced under oxygen limited conditions, suggesting its functional adaptation to dried conditions [1]. It develops from the epidermal cells of the emerging coleorhiza during the early germination process (akin to the root hairs growing form root epidermis), are considered to be responsible for water absorption and could be anchored to the soil during the germination process [2]. Coleorhiza hairs were first reported in germination assays in different rice cultivars including drought resistant upland rice, moderate resistant paddy rice upland and non-resistant rice [1]. The members of *Poaceae* are probably the most valuable plant family including many cereal crops [3]. This specific structure of seeds of *Poaceae* is presumed to have water absorbing properties [4] and may help to exude cohesive-substances into the soil [5].

As a staple food crop consumed worldwide [6], rice (a member of *Poaceae*) feeds more.

than half of the world’s population and provides 20% of daily calories [7]. *Japonica* and *indica* rice are the most cultivated and consumed rice subgroups in China and they have differences in plant architecture, agronomic and physiological features [8]. Coleorhiza hairs also develop on seeds of rice. How rice coleorhiza hairs are formed, and the similarities and differences in coleorhiza hair formation between rice subgroups remain largely obscure.

The rapidly growing world population requires increased production rates of staple crops such as corn and rice but current rice production practices face several obstacles such as global climate change and environmental pollution issues connected to traditional intensive cultivation practices, increasing use of water-land-energy-labor resources, and increasing industrialization and urbanization which leads to the loss of agricultural land area [9]. Hence, in future, a balance between increased rice production and conserving environmental sustainability is needed and can be achieved by adoption of agro-practices consuming less resources in labor, agrochemicals and irrigation water which are also less taxing on the environment [10, 11]. This can be achieved currently by using more efficient rice cultivars which require less resources and by improving agronomic practices such as irrigation management. With respect to labor input reduction, mechanization and direct rice seeding along with weed management are suitable practices already. Environmental footprint for rice production can be reduced by adopting direct seeding practices and costs decreased by using technologies requiring less resources such as alternate wetting and drying irrigation regime [9, 12].

In traditional rice production, in order to save transplant time, labor and costs, farmers directly sow or disperse seeds in flooded fields where seeds germinate in a hypoxic environment which can lead to more greenhouse gas emission [13]. Although the soil preparation before direct seeding has been mechanized for a long time, the surface of rice field is uneven, which means that some places have deep water layer, while some places have no water layer. Therefore, the rice seeds are in different water states (some are completely submerged while some are only partially in contact with water) after direct seeding. We found that under the condition of near saturated air humidity (98%), rice seeds have many coleorhiza hairs. In contrast when the rice seeds are completely submerged in water, there will be no coleorhiza hair formation. We speculate that coleorhiza hair formation plays an important role in improving rice seed germination under water scarce conditions. The study of coleorhiza hair formation may provide new ways thorough which plants could perceive water state and provide theoretical basis for breeding longer (or more) coleorhiza hair varieties which may impart higher germination rate in direct seeding practices.

Omics technologies have been widely used to study plant growth, development and molecular characteristics [14–17], which provides great information and can be used to catalog all the genes expressed in a particular condition [18]. Transcriptome analysis has been widely used for elucidating the expression patterns in the root hair development under multiple environmental conditions in different plant species [19–21]. In this study, a comparative transcriptome analysis was used to address gene regulatory networks in coleorhiza hair regulation of two rice subgroups and it would help improve the understanding of molecular role of coleorhiza hair development in facilitating seed germination.

## Results

### Phenotypic characteristics of coleorhiza hair formation in *japonica* and *indica* rice

**Fig. 1:**
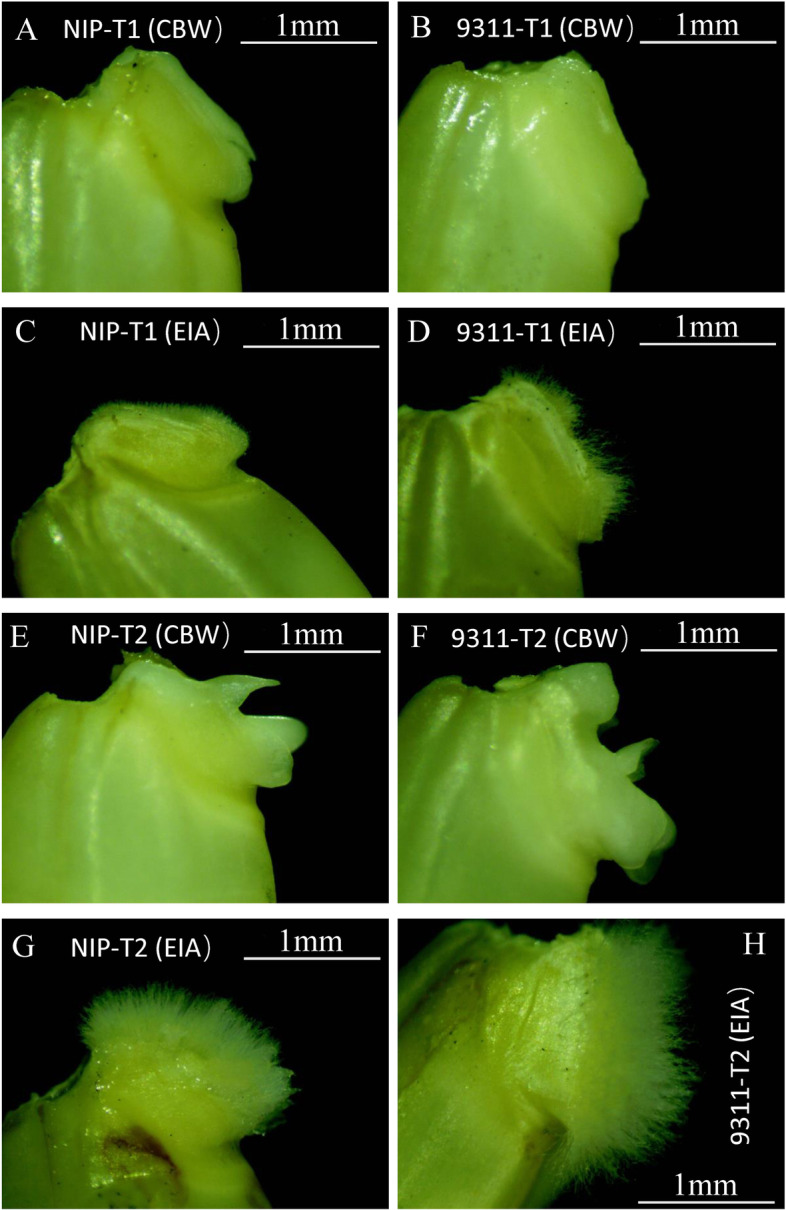
Phenotypic comparison of coleorhiza hair development in *japonica* (NIP) and *indica* (9311) cultivars. **A** and **E**, coleorhiza hair development in *japonica* under CBW at T1 and T2stages. **B** and **F**, coleorhiza hair development in *indica* under CBW at T1 and T2 stages. **C** and **G**, coleorhiza hair development in *japonica* under EIA conditions at T1 and T2 stages. **D** and **H**, coleorhiza hair development in *indica* under EIA conditions at T1 and T2 stages

To investigate the phenotypic differences in coleorhiza development in *japonica* (Nipponbare) and *indica* (9311) cultivars, seeds of these two cultivars were germinated under two water treatments: (a), seeds are fully covered with water, hereby referred to as covered by water (CBW), or (b) seeds are half submerged in water and embryo comes out from the non-submerged part, hereby referred to as embryo in air (EIA). Both CBW and EIA seeds germinated normally within 2 days. Interestingly, the EIA seeds developed a singular phenotype: profuse hair like structures originating from the epidermis of entire embryo surface (coleorhiza, epiblast, and ventral scale) just before root emergence, which were referred to as coleorhiza hairs (Fig. 1). In contrast, no coleorhiza hairs were observed for the CBW treated seeds. Interestingly, although both seeds of Nipponbare and 9311 could develop coleorhiza hairs, coleorhiza hairs of 9311 were significantly longer than those of Nipponbare.

### Difference in type of water treatment was the major factor contributing to transcriptome variation

**Fig. 2:**
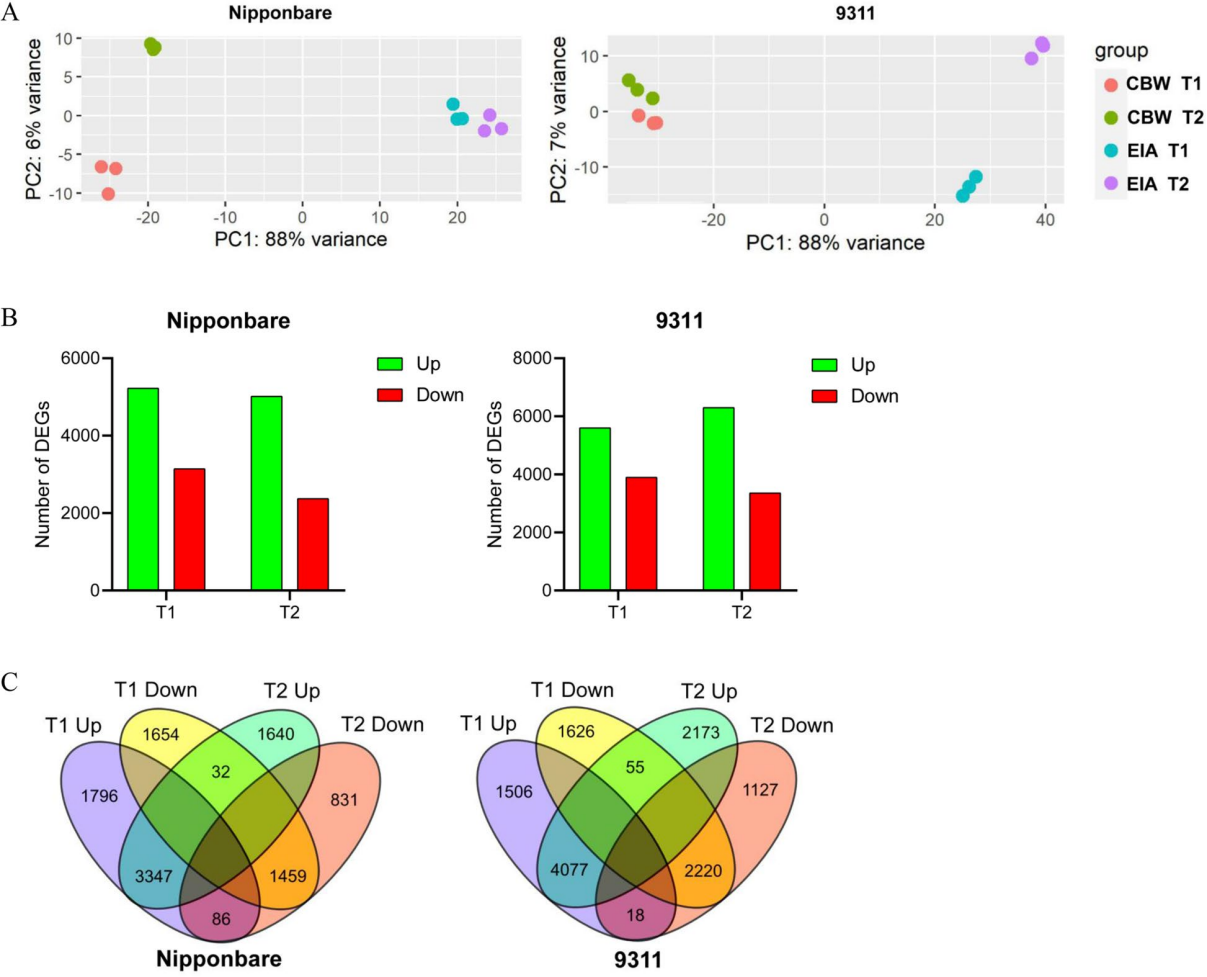
Overview of RNA-seq in coleorhiza hair development of *japonica* and *indica* cultivars. **A**, PCA variation analysis of samples in *japonica* and *indica*. **B**, Total DEGs in *japonica* and *indica* at T1 and T2 stages. **C**, Venn diagram of DEGs shared by *japonica* and *indica* at T1 and T2 stages

To fish out putative genes involved in coleorhiza hair development in Nipponbare and 9311 cultivars during the early germination process, a genome-wide transcriptome approach was employed to compare the EIA and CBW samples of the two rice subgroups. Preliminary data exploration with principal component analysis (PCA) was conducted for the cleaned-up sequencing read data from samples to assess the factors which contributed most to the observed transcriptional variation between samples (Fig. 2A). The replicates for each tissue-treatment combination clustered closely with each other suggesting robust replication in our transcriptome profiling. Along PC1, major variation (88%) was seen because of the treatment (CBW or EIA) while PC2 showed a minor variation (6 & 7%) due to time points. This suggested that differences in water exposure between the two treatments is the major factor affecting the transcriptome variation between treatment samples in the two cultivars. Similarity in transcripts in all samples was assessed by the Pearson Correlation Coefficient (PCC) analysis (Figure S1). Overall, the PCC values ranged from 0.86 to 0.96 (both cultivar at T1 and T2 stages). As expected, the transcripts profiles in Nipponbare and 9311cultivar were highly correlated with each other at T1 and T2 stages. This indicates that the gene expression changes correlate well with each other in both cultivar at early seed germination during coleorhiza hair development process.

### Degree of transcriptome modulation under different treatments and time points

Differences in differentially expressed genes (DEGs) were examined to decipher genes with similar or different regulatory patterns which may participate in coleorhiza hair development under the two treatments. For *japonica* (Nipponbare), 5229 genes were up-regulated, and 3145 genes were down-regulated in T1, and 5019 genes were up-regulated, and 2377 genes were down-regulated in T2 in a comparison of EIA vs CBW samples (Fig. 2B). For *indica* (9311), 5601 genes were up-regulated, and 3901 genes were down-regulated in T1, and 6305 genes were up-regulated, and 3365 genes were down-regulated in T2 in a comparison of EIA vs CBW samples (Fig. 2B). To have an idea of the degree of overlap between these datasets, Venn intersections were carried out between the up-regulated and down-regulated DEGs at the 2 time points for each cultivar separately (Fig. 2C). For Nipponbare, there were 3347 common up-regulated DEGs and 1459 common down-regulated DEGs between T1 and T2. For 9311, there were 4077 common up-regulated DEGs and 2220 common down-regulated DEGs between T1 and T2. The overall DEGs were also analyzed by Volcano plot that displays the consistency between CBW and EIA in both cultivar at T1 and T2 stages (Figure S2). Hierarchical clustering suggested that the treatment effects at both time points lead to similar transcriptional profiles for both cultivars (Figure S3).

### Functional classification of the DEGs by gene ontology (GO) and Kyoto encyclopedia of genes and genomes (KEGG) analysis

To further interpret the genes or metabolic pathways involved in coleorhiza hair development in *japonica* and *indica* cultivars, DEGs identified at early stage (T1) and late stage (T2) of coleorhiza hair formation were subjected to GO enrichment and KEGG pathway analysis. GO enrichment of DEGs from *japonica* (Nipponbare) collected at T1 and T2 stages and from *indica* (9311) collected at T1 and T2 stages were classified into three main GO categories: “cellular component”, “biological processes” and “molecular function” (Figure S4). Not surprisingly, the DEGs from these two time-points of *japonica* and *indica* showed similar GO enrichment terms when compared. In the biological process category, DEGs were mostly enriched in cellular process, metabolic process, response to stimulus and biological regulation (at both T1 and T2). In the cellular component category, cell part, organelle, membrane part, and membrane were significantly enriched terms (at both T1 and T2). In the molecular function category, the top four enriched molecular function terms were binding, catalytic activity, transporter activity and transcription regulator activity (at both T1 and T2).

**Fig. 3:**
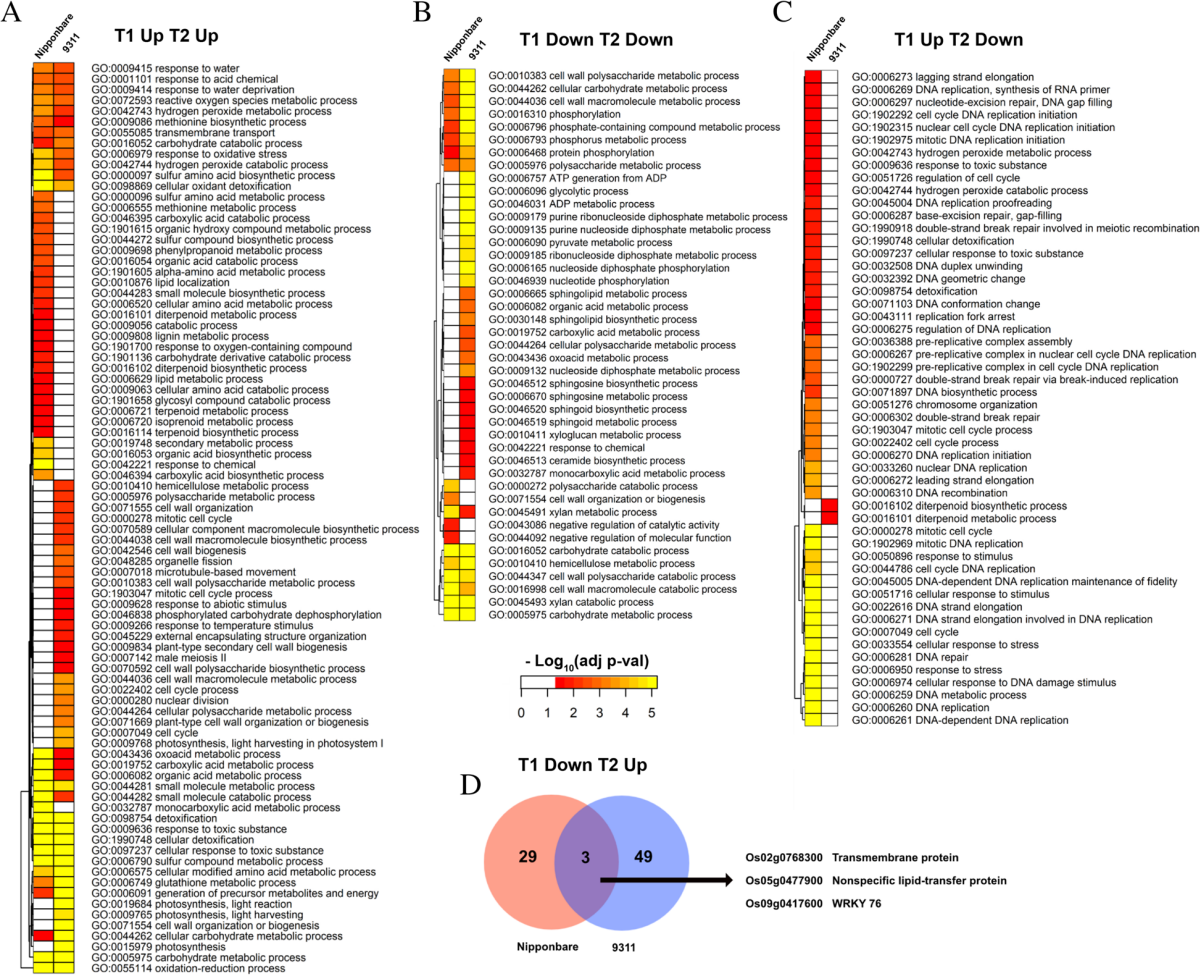
Biological process (BP) in GO enrichments analysis of DEGs in *japonica* and *indica*. **A**, Up-regulated DEGs at T1 and T2 enriched in BP in both cultivars. **B**, Down-regulated DEGs at T1 and T2 enriched in BP in both cultivars. **C**, DEGs up-regulated at T1 and down-regulated at T2 enriched in BP in both cultivars. **D**, Common DEGs down-regulated at T1 and up-regulated at T2 stage in both cultivars

Furthermore, specifically, using GO comparison heatmaps, the enriched “biological process” (BP) terms for DEGs up-regulated at T1 and T2, up-regulated at T1 but down-regulated at T2, and down regulated at both T1 and T2 in these two cultivars were analyzed respectively for finding similar or distinct processes active at these time points during coleorhiza development (Fig. 3). There are about 34 common enriched BP terms including “response to water”, “responses to acid chemical”, “response to water deprivation”, etc. for DEGs up-regulated at both T1 and T2 in these two cultivars. Subsequently, there are 23 unique BP terms such as “secondary metabolic process”, and “organic acid biosynthetic process”, enriched only in *japonica* (Nipponbare), and 19 BP terms including “cell wall organization”, “cell wall biogenesis”, etc., enriched specifically in *indica* (9311) (Fig. 3A). Secondly, 15 BP terms are commonly enriched for DEGs down-regulated at both T1 and T2 stages in *japonica* and *indica*, 4 BP terms (“polysaccharide catalytic process”, “cell wall organization or biogenesis”, “negative regulation of catalytic activity”, “negative regulation of molecular function”) are specifically enriched only in *japonica* cultivars and 24 BP terms including “ATP generation from ADP”, “glycolytic process”, “ADP metabolic”, etc., only in *indica* cultivars (Fig. 3B). Thirdly, 52 BP terms were enriched for DEGs up-regulated at T1 and down-regulated at T2 out of which 50 were specific to *japonica* and 2 (“diterpenoid biosynthetic process” and “diterpenoid metabolic process”) specific to *indica* (Fig. 3C). There are 3 DEGs (“WRKY”, “transmembrane protein”, “lipid transfer protein”) that are down-regulated at T1 and up-regulated at T2 in both *japonica* and *indica*, (Fig. 3D).

**Fig. 4:**
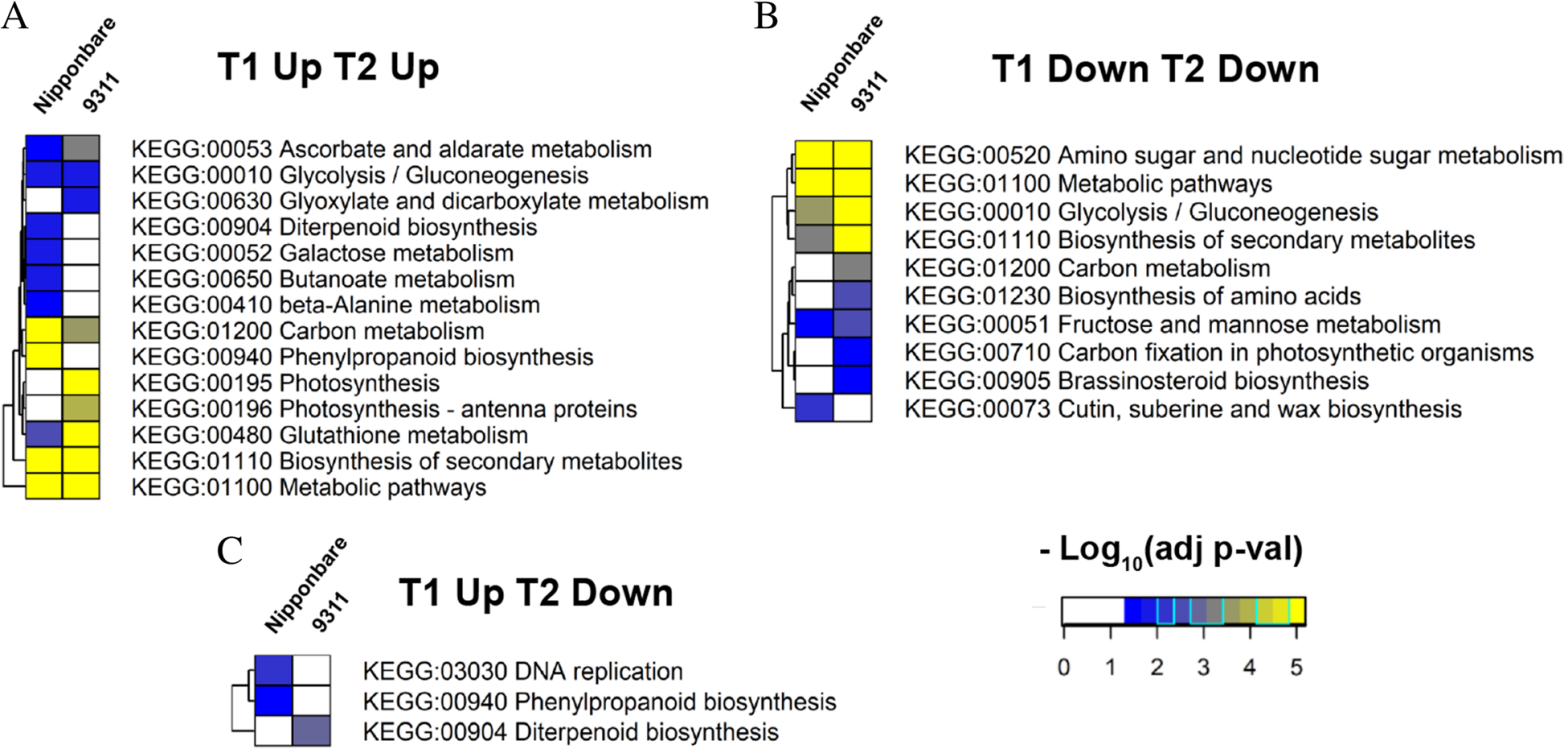
KEGG analysis of DEGs accumulated pathways shared in *japonica* and *indica*. **A**, DEGs that were up-regulated at T1 and T2 stages in accumulated pathways. **B**, DEGs that were down-regulated at T1 and T2 stages in accumulated pathways. **C**, DEGs that were up-regulated at T1 stages and down-regulated at T2 stages in accumulated pathways

To identify the specific metabolic pathways perturbed in the treatment comparison for the two time points in both cultivars, KEGG pathway enrichment analysis was employed. The top 12 KEGG pathways involving most DEGs (Figure S5) included carbon metabolism, phenylpropanoid biosynthesis, biosynthesis of amino acids, plant hormone signal transduction, starch and sucrose metabolism, glycolysis/gluconeogenesis, amino sugar and nucleotide sugar metabolism, plant-pathogen interaction, glutathione metabolism and MAPK signaling pathway-plant were enriched for the two rice cultivars in both T1 and T2. To further discern the metabolic pathways that may be common or distinctive for these two cultivars, KEGG comparison heatmaps were visualized. Among upregulated DEGs, four distinctive pathways including diterpenoid biosynthesis, galactose metabolism, butanoate metabolism, beta-alanine metabolism were found enriched for DEGs up-regulated at T1 and T2 specifically in *japonica*, while three distinctive pathways including glyoxylate and dicarboxylate metabolism, photosynthesis and antenna proteins enriched for DEGs up-regulated at both T1 and T2 specifically in *indica* and six pathways were commonly enriched for both cultivars for DEGs up-regulated at both T1 and T2 (Fig. 4A). Among downregulated DEGs, enriched metabolic pathways for DEGs down-regulated at both T1 and T2 in both cultivars were amino acid and nucleotide sugar metabolism, metabolic pathways, glycolysis/gluconeogenesis, and biosynthesis of secondary metabolites. Other KEGG terms specific to *indica* were carbon metabolism, biosynthesis of amino acids, carbon fixation in photosynthetic organism, and brassinosteroid biosynthesis while fructose and mannose metabolism and cutin, suberin and wax biosynthesis were specifically enriched for *japonica* (Fig. 4B). Among contrastingly regulated DEGs, DNA replication and phenylpropanoid biosynthesis are enriched for DEGs up-regulated at T1 and down-regulated at T2, specifically for *japonica* and diterpenoid biosynthesis specifically for *indica* (Fig. 4C).

### Genes involved in water deprivation and carbohydrate catabolism were upregulated during coleorhiza hair formation

**Fig. 5:**
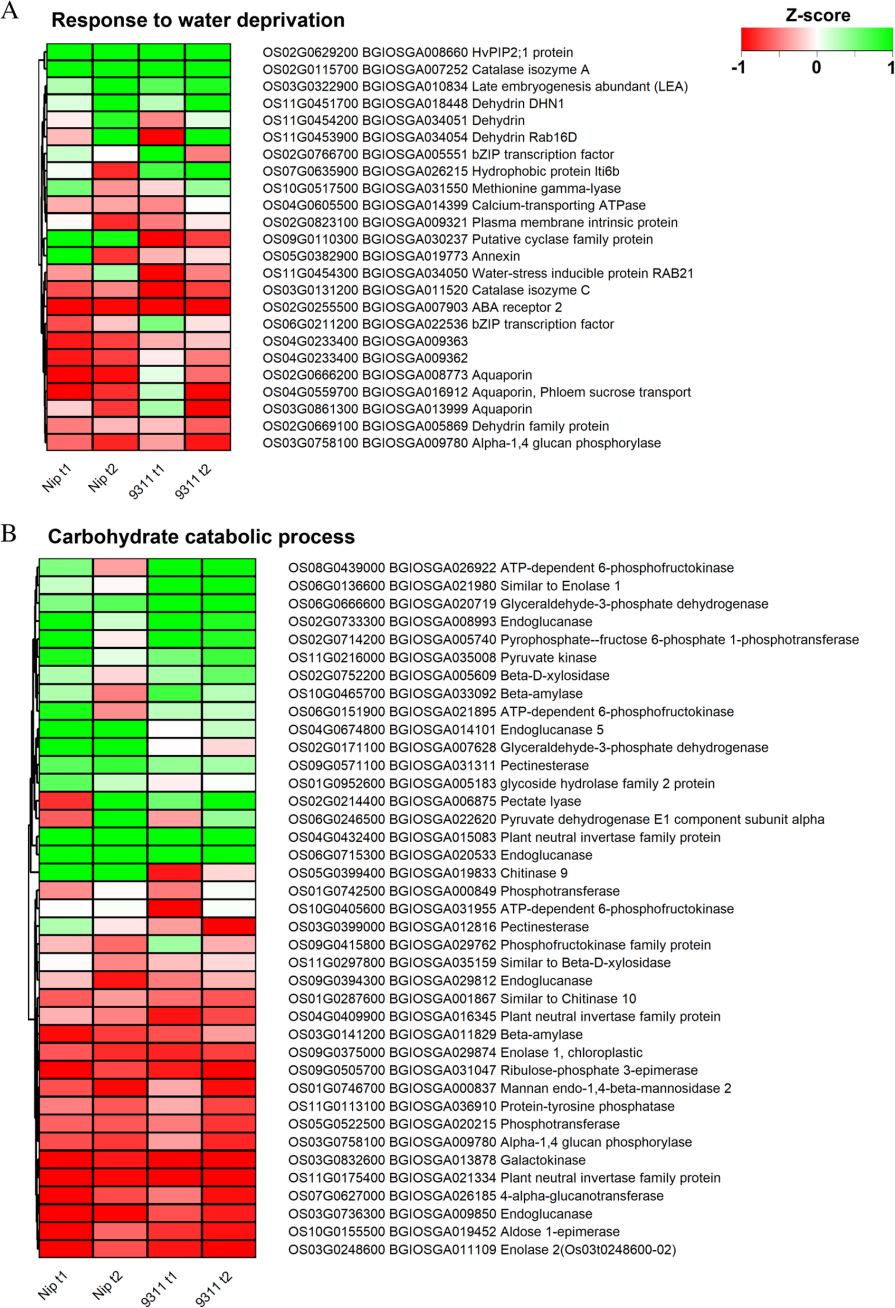
DEGs involved in GO enriched biological processes. **A**, DEGs enriched in “responses to water deprivation” BP categories. **B**, DEGs enriched in “carbohydrate catabolic process” BP categories

**Fig. 6:**
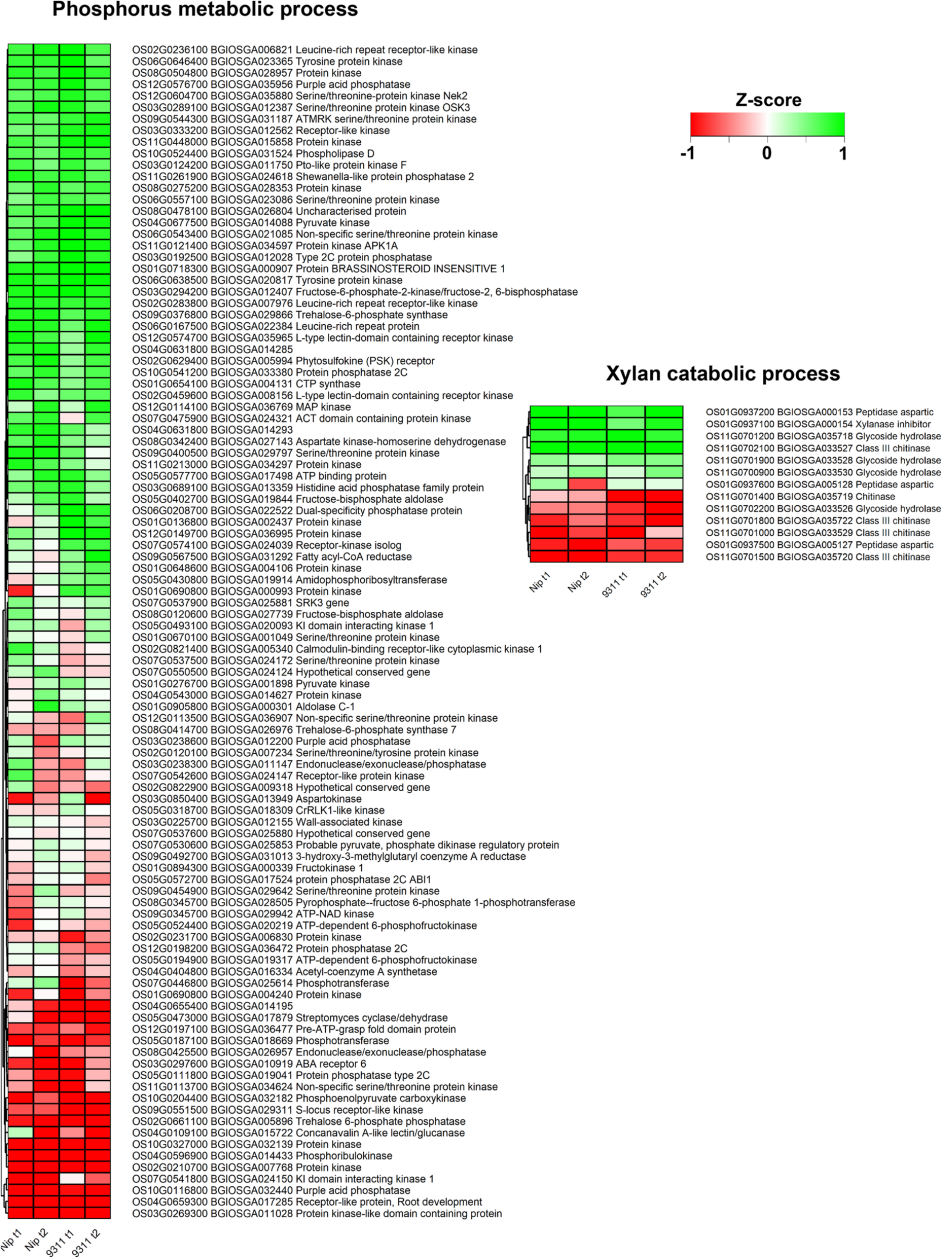
DEGs enriched in “phosphorus catabolic process and xylan catabolic process” BP categories of GO enrichments

To further investigate the specific DEGs that may be involved in coleorhiza development of *japonica* and *indica* cultivars, expression differences between *japonica* and *indica* were analyzed. Firstly, DEGs constituted in the enriched BP terms “response to water deprivation” and “carbohydrate catabolic process” for DEGs upregulated at T1 and T2 (Fig. 5); “glutathione metabolic process, response to oxidative stress, hydrogen peroxide catabolic process”, and “carbon metabolism” for DEGs upregulated at T1 and T2 (Figure S4); “phosphorus metabolic process” and “xylan metabolic process” for DEGs downregulated at T1 and T2 (Fig. 6) were compared for log_2_ fold change differences between the cultivar samples (Figs. 5, 6, S4). Secondly, KEGG term DEGs for “Carbon metabolism”, “Glutathione metabolism”, “Amino acid sugar metabolism”, “nucleotide sugar metabolism”, “fructose and mannose metabolism” were also analyzed for expression differences between the analyzed samples (Figure S5, S6). Log_2_ fold changes were scaled to Z scores between the samples for DEGs constituted in all the above terms.

DEGs constituting “response to water deprivation” (Fig. 5A) and “carbohydrate catabolic process” (Fig. 5B) were compared for expression differences between *japonica* and *indica*. In total, 24 DEGs in “response to water deprivation” were upregulated commonly in both cultivars and at both time points. Among these genes, transcript levels of four genes encoding phosphatidylinositol 4,5-bisphosphate (PIP2, *Os02g0629200*/*BGIOSGA008660*), catalase isozyme A (*Os02g0115700*/*BGIOSGA007252*), later embryogenesis abundant (*LEA*, *Os03g0322900*/*BGIOSGA010834*), and dehydrin (*DHN1*, *Os11g0451700*/*BGIOSGA018448*) respectively were expressed similarly in both cultivars and at both time points. Two dehydrin genes (*Os11g0454200*/*BGIOSGA034051*, *Os11g0453900*/*BGIOSGA034054*) and genes encoding water stress inducible protein RAB21 (*Os11g0454300*/*BGIOSGA034050*) in both cultivars were induced at T2 compared to T1. One bZIP transcriptional factor gene (*Os02g0766700*/*BGIOSGA005551*) showed down-regulation at T2 in *japonica* and *indica* as compared with T1 while other bZIP (*Os06g0211200*/*BGIOSGA022536)* increases at T2 in *japonica* but decreases at T2 in *indica*. Three aquaporin genes (*BGIOSGA008773*, *BGIOSGA016912 BGIOSGA013999*) in *indica* had lower expression at T2 as compared to T1. Expression of another gene encoding a hydrophobic protein lti6b (*Os07g0635900*/*BGIOSGA026215*) was reduced at T2 in *japonica* but remained to similar levels at T1 and T2 in *indica.* A methionine gamma-lyase gene (*Os10g0517500*/*BGIOSGA031550*) and annexin gene (*Os05g0382900*/*BGIOSGA019773*) shows contrasting trends between cultivars. Expression of a calcium-transporting ATPase gene (*Os04g0605500*/*BGIOSGA014399*) increased at T2 in *indica* only. A cyclase family protein gene (*Os09g0110300*/*BGIOSGA030237*) had higher expression at both T1 and T2 in *japonica* relative to *indica*. A catalase isozyme C gene (*Os03g0131200*/ *BGIOSGA011520*) and abscisic acid (ABA) receptor 2 gene (*Os02g0255500/ BGIOSGA007903*) were expressed to similar levels in all samples. A glucan phosphorylase gene (*Os03g0758100*/*BGIOSGA009780*) was repressed at T2 in both cultivars. Comparing expression among genes, dehydrin genes were more upregulated than aquaporin genes.

Interestingly, there are 40 genes involved in the carbohydrate catabolic process were upregulated commonly in both cultivars and at both time points. However, five genes encoding ATP-dependent 6-phosphofructokinase (*Os08g043900*, *Os06g0151900*), beta-D-xylosidase (*Os02g0752200*) and beta-amylase (*Os10g0465700*) were expressed at lower levels at T2 vs. T1 in *japonica* specifically. A gene encoding a pectate lyase (*Os02g0214400*) in *japonica* specifically and a pyruvate dehydrogenase E1 subunit (*Os06g0246500*/*BGIOSGA022620*) in both cultivars were expressed at lower levels at T1 vs. T2.

### Genes involved in phosphorus metabolism and xylan catabolism were downregulated during coleorhiza hair formation

Based on the GO enrichment analysis, genes involved in the phosphorus and xylan catabolic processes were commonly downregulated in both cultivars at both time points T1 and T2 (Fig. 6). In our study, there are 114 DEGs in phosphorus metabolic process and 14 DEGs in xylan catabolic process which are significantly downregulated at T1 and T2 in both cultivars. These DEGs that are involved in phosphorus metabolism most are slightly downregulated. Most DEGs in xylan catabolism are very similarly downregulated in all the samples.

### Glutathione metabolism, hydrogen peroxide catabolic, carbon metabolism, amino and nucleotide metabolism, and fructose and mannose metabolism showed significant transcript changes during coleorhiza hair formation

DEGs mapped to the GO and KEGG terms in glutathione metabolism, reactive oxygen species (ROS) oxidative stress, hydrogen peroxide catabolic, carbon metabolism (for DEGs upregulated at both T1 and T2 in both cultivars) and amino and nucleotide sugar metabolism, and fructose and mannose metabolism (for DEGs downregulated at both T1 and T2 in both cultivars) were analyzed for expression differences in *japonica* and *indica*. For glutathione metabolism, most of the genes belonged to peroxidase encoding genes and glutathione-S-transferase encoding genes (Figure S6). Term carbon metabolism (Figure S7), constituted of genes encoding enzymes such as catalase isozyme A (*Os02g0115700*/*BGIOSGA007252*), alcohol dehydrogenase (*Os07g0621800*/*BGIOSGA023891*), glyceraldehyde-3-phosphate dehydrogenase (*Os06g0666600*/*BGIOSGA020719*), enclose1 (*Os06g0136600*/*BGIOSGA021980*), alcohol dehydrogenase (*Os03g0189600*/*BGIOSGA011308*), phosphoenolpyruvate carboxylase (*Os08g036600*/*BGIOSGA027083*), pyruvate kinase (*Os11g0216000*/*BGIOSGA035008*), crotonase (*Os06g0594100*/*BGIOSGA023191*) and AMP-binding protein (*Os03g0305000*/*BGIOSGA010894*). DEGs commonly downregulated in both cultivars at T1 and T2 and involved in amino acid metabolism belonged to chitinases (Figure S8).

### Genes involved in ABA and auxin signaling pathways were up-regulated during coleorhiza hair formation

**Fig. 7:**
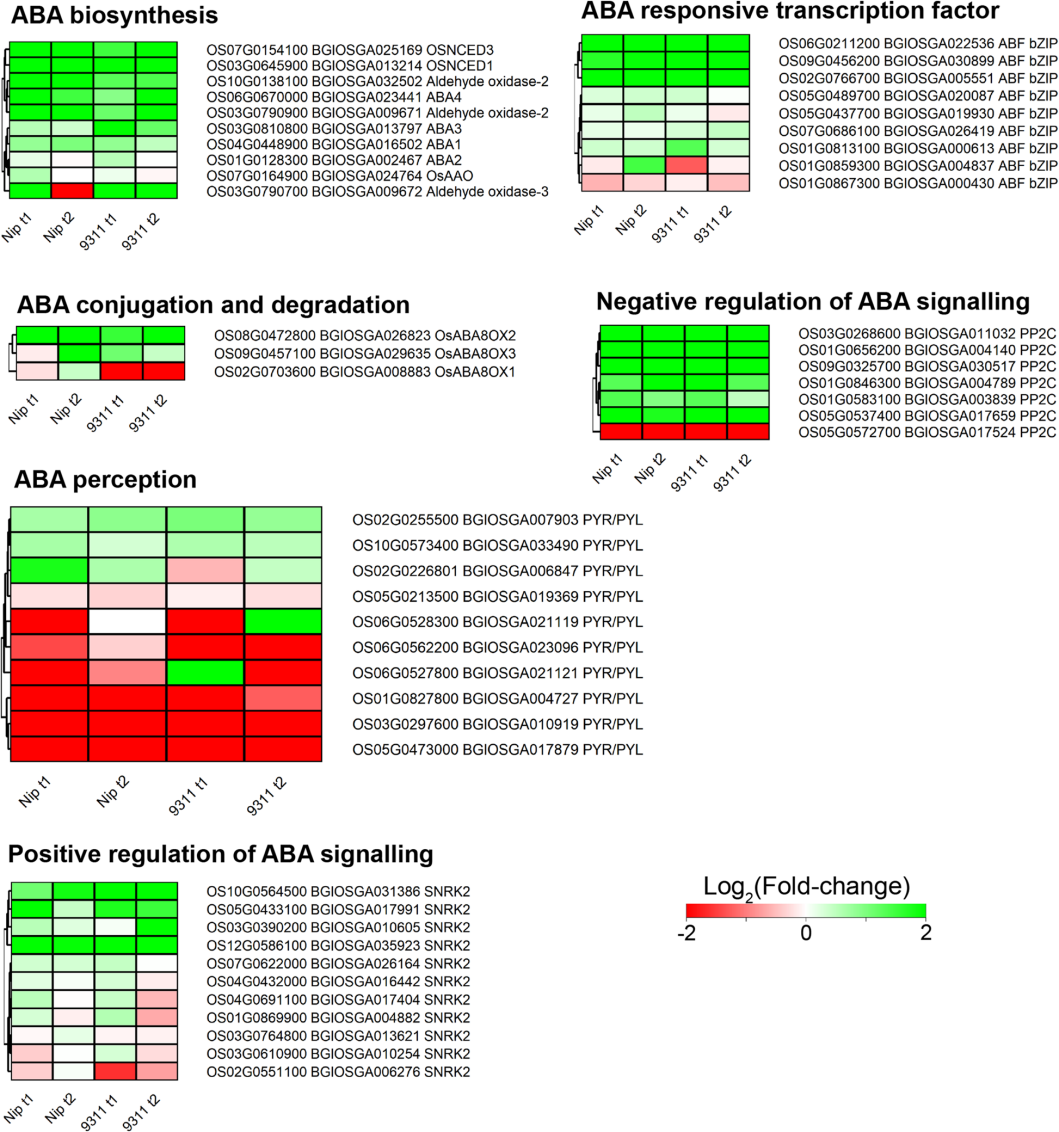
KEGG analysis of DEGs related to ABA biosynthetic pathways in coleorhiza hair development in *japonica* and *indica*

To identify the genes in the ABA signaling pathway, the DEGs were mapped against the KEGG pathway. Most genes involved in ABA synthesis, ABA responsive transcriptional factor, ABA conjugation and degradation and in negative or positive regulation of ABA signaling were up-regulated (Fig. 7). However, the ABA receptor genes displayed mixed expression patterns and six receptor genes (*PYLs*, *Os05g0473000*/*BGIOSGA017879*, *Os03g0297600*/*BGIOSGA010919*, *Os01g0827800*/*BGIOSGA004727*, *Os06g0527800*/*BGIOSGA021121*, *Os06g0562200*/*BGIOSGA023096*, *Os05g0213500*/*BGIOSGA019369*) were down-regulated while three receptor genes (*Os02g0255500*/*BGIOSGA007903*, *Os10g0573400*/*BGIOSGA033490*, *Os02g0226801*/*BGIOSGA006847*) were up-regulated in both cultivar at T1 and T2 stages (Fig. 7).

**Fig. 8:**
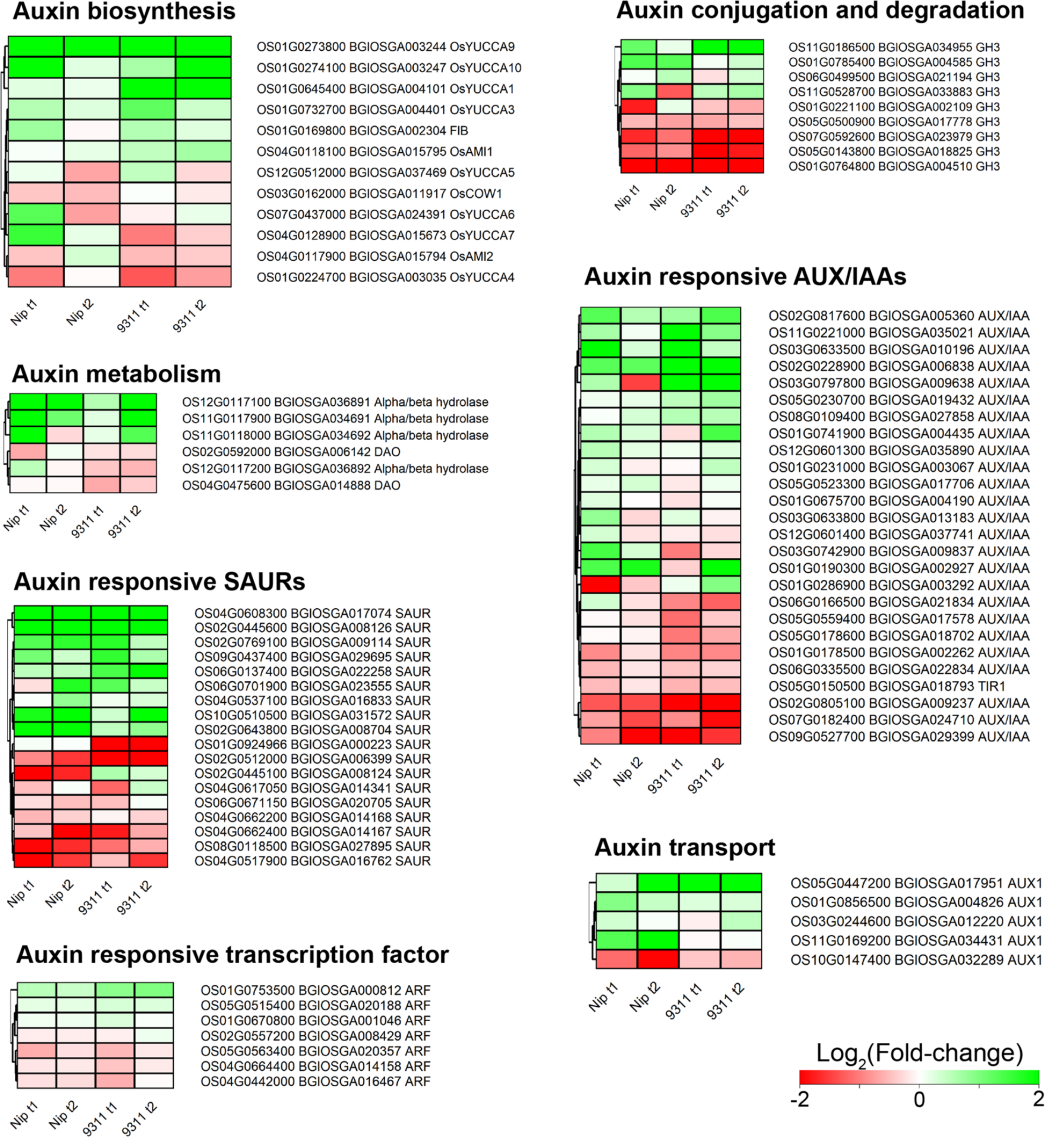
KEGG analysis of DEGs related to auxin biosynthetic pathways in coleorhiza hair development in *japonica* and *indica*

Since auxin has been implicated in root hair formation, DEGs involved in the auxin related pathways were analyzed. In our study, DEGs involved in auxin biosynthesis, auxin transport, auxin metabolism, and auxin response were greatly up-regulated while most of the genes belonging to auxin conjugation and degradation were down-regulated (Fig. 8). However, the auxin responsive TFs were slightly down-regulated and only two of them (*Os01g0753500*/*BGIOSGA000812*, *Os05g0515400*/*BGIOSGA020188*) were up-regulated in both cultivar at T1 and T2 stages (Fig. 8).

### Gene expression changes in quantitative real-time PCR (qRT-PCR) and RNA-Seq assays are strongly correlated

Fifteen genes belonging to ABA and auxin signaling pathways and response to water deprivation pathway were selected for gene expression verification (Figure S9). The expression fold changes from qRT-PCR strongly correlated with those obtained from RNA-Seq, with a correlation coefficient of *R*^*2*^ = 0.865, indicating that the gene expression changes obtained with RNA-Seq were robustly obtained.

## Discussion

The coleorhiza, a non-vascularized embryonic organ that expands upon imbibition, emerges from the radicles and is thought to play essential roles in protecting growing embryo and controlling the germination [22]. It is a root hair-like structure, starting elongation from the coleorhiza of the developing embryo during the early germination process and is only present under the oxygen-limited and moisture dependent conditions in rice [1, 2]. These are thought to supply water to the embryo during germination [23, 24]. Afterwards, the coleorhiza hair, reported to function as an anchor, could adhere into the soil in a direct penetration ratio of seminal root in the surface-sown forage grass seeds [2]. This coleorhiza hair is not simply an adventitious outgrowth as validated by the failure of sub-surface cells to produce them but also work as an important organ to help the plant survival when exposed to stresses [2]. However, few functional developments have been made since the discovery of the coleorhiza hair [25]. Nevertheless, it is quite intriguing to elucidate the molecular basis of coleorhiza hairs in germinating seeds and its responses to various environmental conditions. Unlike coleorhiza hair, root hair is a tubular-shaped outgrowth of root epidermis cells but differentiate only during the late stage of plant growth [26, 27]. Previously, large scale transcriptome analysis had been performed to investigate the responses of root hairs to water deficiency, excessive water conditions as well as nutrients, hormone, stress and etc., demonstrating that root hair are of vital importance to regulate the stress adaptations and maintain the basic plant development [28–30]. However, fewer studies have been performed to investigate the gene regulatory networks in coleorhiza hair development in seed germination at an early stage.

In this study, we performed a comparative transcriptome analysis of gene expression in coleorhiza hair development of *indica* (9311) and *japonica* (Nipponbare) rice cultivars under EIA and CBW conditions at the early stage of germination. These are most cultivated varieties and parental lines used for breeding in Asian countries [31]. Phenotyping of embryos in air vs fully submerged embryos at 12 and 24 h (T1 and T2) post sowing in two rice cultivars *japonica* and *indica* demonstrated that *indica* (9311) has longer coleorhiza hairs at both T1 and T2 as compared to *japonica* (Nipponbare) under EIA as compared to CBW (Fig. 1). A subsequent genome-wide transcriptome analysis on these embryos suggested that more DEGs are identified in *indica* (9311) than in *japonica* (Nipponbare) with EIA treatment at T1 and T2 stages (Fig. 2-b). Specifically, there are 3347 DEGs that were commonly detected as up-regulated genes at T1 and T2 while there are 1459 DEGs that were commonly detected as down-regulated at T1 and T2 in *japonica* (Fig. 2-c). Again, from these some DEGs were up-regulated (86) at T1 and down-regulated at T2 as well as some DEGs down-regulated at T1 and up-regulated at T2 (32) (Fig. 2-c). Meanwhile, the comparison in *indica* (9311) indicated that the number of common up-regulated DEGs at T1 and T2 is about 4077 and common down-regulated DEGs at T1 and T2 is about 2173. On the other hand, some DEGs were up-regulated (18) at T1 and down-regulated at T2 while some DEGs were down-regulated at T1 and up-regulated at T2 (55) (Fig. 2-d).

To interpret the specific DEGs that may be responsive in coleorhiza hair development, the common DEGs identified from two cultivars at T1 and T2 stages were compared and analyzed via GO enrichment and KEGG mapping (Fig. 3 and Fig. 4). Interestingly, the “biological process” in the GO category of the shared common DEGs revealed that DEGs enriched in “response to water”, “responses to water deprivation”, “reactive oxygen species metabolic process” and “carbohydrate catabolic process” were up-regulated in both *japonica* and *indica* while the DEGs enriched in biological process such as “cell wall related metabolism process”, “phosphorus metabolic process”, and “xylan catabolic process” were down-regulated in both cultivars. However, the DEGs enriched in “diterpenoid biosynthetic and catabolic process” were up-regulated at T1 and down-regulated at T2 in both cultivars. There were about 22 upregulated DEGs related to “responses to water deprivation”, suggesting that those genes are specific responsive genes to water signal that could regulate coleorhiza hair development in both *japonica* and *indica* cultivars. For example, aquaporin family genes (*PIP1–1, Os02g0666200; PIP1–2, Os04g0559700; PIP2–8, Os03g0861300*), are a family of integral membrane associated water channel proteins functioning for the water transport and small molecules [32], and were upregulated in both cultivars at T1 and T2 stages. *OsPIP1–1*, is induced by drought and salt stress, and its overexpression could increase the resistance to water and stress which also improves the seed germination, root hydraulic conductivity and seed yield [33]. PIP1–2 was reported to be involved in the mesophyll CO_2_ and sucrose transport [34]. However, the function of PIP2–8 needs to be established [35]. Thus, upregulation of aquaporin genes in EIA samples may enhance water uptake to impart tolerance to water deficiency for optimal seed germination as compared to the seeds under CBW conditions. Another group of DEGs, involving dehydrin family genes (*Os11g0451700, Os11g0453900, Os11g0454200*) are mostly up-regulated in EIA samples at T1 and T2 stages in both cultivars (Fig. 3). The dehydrin family genes belong to a subgroup of the late embryogenesis abundant proteins and gene expression is highly up-regulated by multiple stress conditions including drought, cold and salt [36]. Undoubtedly, the seeds of *japonica* and *indica* treated under EIA may suffer from drought stress which in turn could cause up-regulation of dehydrin genes and help improve the growth of coleorhiza hair to cope up with drought onset in both cultivars. These results prove that the coleorhiza hair development may be essential for uptake of water and alleviation of drought stress under EIA conditions in both cultivars. KEGG analysis of the DEGs common to both cultivars suggested that biosynthesis of secondary metabolites, glutathione metabolism, carbon metabolism and glycolysis/gluconeogenesis were greatly enriched in upregulated DEGs in both cultivars at T1 and T2 stages (Fig. 4, Figure S5). In addition, nearly half of the DEGs involved in glutathione metabolism and carbon metabolism were upregulated and DEGs involved in amino acid and nucleotide sugar metabolism were downregulated under EIA conditions in both cultivars (Figure S6-S7). Glutathione metabolism plays important roles in stress tolerance of plants to many abiotic stresses including salt, drought, chilling as well as plant development [37, 38] and thus its upregulation would prepare germinating seeds with better tolerance to oxidative stress by quenching ROS. Previous studies indicated that up-regulation of genes in carbon metabolism would lead to changes in level of sugars in plants including those of glucose and sucrose that are generally correlated to universal stress conditions and would help supply energy for plant root development during germination process [39]. Similarly, as for KEGG analysis, glutathione metabolism and carbon metabolism related genes are induced in coleorhiza hair containing embryos under EIA as compared to CBW treated ones which are fully covered by water i.e., experiencing flooding and low oxygens stress. Summarizing, coleorhiza hair may help improve survival of imbibed seeds under oxidative stress and avail energy from carbon metabolism but formation of higher metabolites like amino acid sugar or nucleotide sugar are not pursued during this process. This would suggest that more focus is put on the growth processes of seed germination rather than producing more metabolic intermediates and energy for this growth comes from the endosperm energy reserves. Carbohydrate catabolic process was related to oxygen deprivation in terms of growth and survival of plant organs [40]. In rice, carbohydrate metabolism plays vital roles in sustaining coleoptile elongation [41]. As seed storage substances such as carbohydrates, lipids and proteins can be mobilized into embryo to fuel the seed germination process [42], up-regulation of genes encoding enzymes in pathways like carbohydrate catabolic process may enhance coleorhiza hair development as observed in our study. Consistent with this hypothesis, the DEGs involved in carbohydrate metabolism were mostly up-regulated that may help maintain the coleorhiza hair development in *japonica* and *indica* cultivars (Fig. 5-b). It is quite intriguing that DEGs participating in phosphorus metabolic process are downregulated in coleorhiza hair development in both cultivars under EIA conditions (Fig. 6) which may suggest preference of growth over biosynthesis of metabolites.

Hormones such abscisic acid (ABA) and gibberellin (GA) are thought to be the main hormones regulating seed germination in plants [43]. Abscisic acid (ABA) also plays a vital role in root hair elongation [44] and is involved in inhibition of seed germination [45]. However, recent reports also addressed another plant hormone, auxin, which is also critical for inducing and maintaining seed dormancy and may act as key regulator for protecting seed dormancy [46]. This suggests that seed germination may be regulated by phytohormones. However, in our study, the GA metabolism related genes were not significantly expressed in both cultivars in *japonica* and *indica* and only ABA and auxin related genes were mainly upregulated in both cultivars at both T1 and T2 (Fig. 7 and Fig. 8). Auxin emerged as a regulator together with ABA to regulate seed germination and induction and maintenance of seed dormancy [43]. Auxin is also involved in root hair formation [47–49] and thus it is tempting to assume that coleorhiza hair formation is also regulated by auxin in conjunction with ABA. Interestingly, free auxin depleting enzyme genes which are responsible for auxin conjugation and degradation are downregulated. The upregulated genes in other auxin processes can be functionally analyzed using genetic and molecular biology experiments during coleorhiza hair development.

Overall, this is the first study comparing transcriptomic responses in coleorhiza hair development in two major rice cultivars, *japonica* and *indica* during seed germination process and hypothesize the putative role of common transcriptionally perturbed genes and metabolic pathways in coleorhiza hair containing embryos of two different rice cultivars in mediating coleorhiza hair development. This paves way for detailed functional studies in coleorhiza hair developmental biology in the future.

## Conclusions

In this study, seeds of *japonica* variety Nipponbare and *indica* variety 9311 could develop coleorhiza hairs under EIA treatments, and coleorhiza hairs of 9311 were significantly longer than those of Nipponbare. There are differences in DEGs quantity and enriched pathways between Nipponbare and 9311, which may lead to different coleorhiza hair length. DEGs enriched in water deprivation, ABA and auxin metabolism, carbohydrate catabolism and phosphorus metabolism in both two varieties, which may play important roles in coleorhiza hair formation.

## Methods

### Plant materials and growth conditions

Seeds of a *japonica* Nipponbare and an *indica* 9311 cultivars were kindly provided by Prof. Jianchang Yang (Jiangsu Key Laboratory of Crop Cultivation and Physiology, Yangzhou University, China). Ripened seeds of cultivar Nipponbare and cultivar 9311 were dried in an oven at 50 °C for three days to break seed dormancy. Hulled rice seeds were first glued to the middle of slides (10 seeds/slide), and 6 slides were placed in one slide box. After that, these seeds were germinated under two treatments. Treatment one: half of seed surface was in water, and the rest half with embryo in air (EIA) and treatment two: whole seeds were covered by water (CBW). Each treatment contained 3 boxes (18 slides) for one replicate for one cultivar. Seeds were placed in an incubator with the temperature maintained at 28 ± 5 °C and kept in dark.

### RNA extraction, RNA sequencing, data analysis, and quality control of RNA-seq

EIA and CBW treated Nipponbare and 9311 RNA samples (embryo) were collected first, when coleorhiza hairs just developed in EIA treatment (about 24 h after treatment, T1), and second, when coleorhiza hairs were the longest (about 36 h after treatment, T2). RNA was extracted using E.Z.N.A.® plant RNA Kit (Omega Bio-tek, GA, USA) and quantified with kaiaoK5500®Spectrophotometer (Kaiao, Beijing, China). RNA integrity and concentration were assessed using RNA Nano 6000 Assay Kit in Bioanalyzer 2100 (Agilent Technologies, CA, USA). RNA concentration for library preparation was measured with Qubit® RNA Assay Kit in Qubit® 3.0 and then diluted to 1 μg/μl.

### Library preparation for RNA sequencing

2 micrograms of total RNA was input to NEBNext® Ultra™ RNA Library Prep Kit for Illumina® (NEB, USA) to generate sequencing libraries as follows: poly-T oligo-attached magnetic bead purification of mRNA from input total RNA; mRNA fragmentation by adding divalent cations under heating; first strand cDNA synthesis was performed with random hexamer primers; RNAse H degradation of residual RNA; second strand cDNA synthesis and purification with QiaQuick PCR kit followed by terminal repair, A-tailing and adapter addition. PCR was performed to finish library preparation.

### Library examination, clustering, and sequencing

Insert size in library was quantified with StepOnePlus™ Real-Time PCR System (Library valid concentration > 10 nM). The cBot cluster generation system was used for sample clustering using HiSeq PE Cluster Kit v4-cBot-HS (Illumina, USA). Libraries were sequenced on Illumina platform and 150 bp paired-end reads were obtained for further transcriptome data analysis.

### Data assembly and transcriptome analysis

Quality check of above obtained reads was carried out with fastqc and trimming/adaptor removal was carried out to obtain clean reads. Basic statistics of total raw and clean reads of transcriptome sequencing is shown in Table S1. HISAT2 v2.0.5 was used to perform alignments of bisulfite-treated reads to the reference genome in RAP-DB (https://rapdb.dna.affrc.go.jp/download/irgsp1.html) and Ensembl Plants (http://plants.ensembl.org/Oryza_indica/Info/Annotation/#assembly) using default parameters. Following this, read count for each gene in each sample was obtained with HTSeq v0.6.0, and post normalization of read counts, FPKM (Fragments Per Kilobase Million mapped reads) was calculated to estimate the expression level of genes in each sample. Mapping statistics of clean reads of RNA sequencing data to reference rice genome is shown in Table S2. DESeq2 v1.6.3 was used for differential gene expression by estimating the gene expression level by linear regression calculating the fold changes for sample comparisons; *p*-value with Wald test and corrected *p*-value (q-value) following BH adjustment. Genes with q ≤ 0.05 and |log_2__fold change| ≥ 1 were identified as differentially expressed genes (DEGs). The global gene expression pattern between all samples was denoted by Pearson correlation matrix for calculation of pairwise correlation coefficient (Figure S1) while volcano plots were used for visualizing the distribution of differentially expressed genes (DEGs) (Figure S2); read counts of DEGs in all samples were clustered using hierarchical clustering of normalized counts (Figure S3). Functional enrichment analyses were performed using GO enrichment and KEGG pathway analyses.

### qRT-PCR validation

Validation of the RNA-Seq results was performed for 15 genes using qRT-PCR, according to the method described in Song et al. [17]. The primer sequences used for qRT-PCR are provided in Table S3.

## Supplementary Information

**Additional file 1: Fig. S1.** Correlation matrix heatmap visualizing the Pearson Correlation coefficient between RNA-Seq samples. **Fig. S2.** Volcano plot of differentially expressed genes (DEGs) for EIA vs. CBW treated samples. **Fig. S3.** Hierarchical clustering heatmap between RNA-Seq samples. **Fig. S4.** Overview of GO enrichment analysis of DEGs in *japonica* and *indica* from T1 and T2 stages. **Fig. S5**. Overview of top 10 KEGG pathways f DEGs in *japonica* and *indica* from T1 and T2 stages. **Fig. S6.** DEGs enriched in “glutathione metabolic process, response to oxidative stress, hydrogen peroxide catabolic process” BP categories of GO enrichments. **Fig. S7.** DEGs enriched in “carbon metabolism process” BP categories of GO enrichments. **Fig. S8**. DEGs enriched in “amino sugar and nucleotide sugar metabolism and fructose and mannose metabolism” BP categories of GO enrichments. **Fig. S9**. Comparison of the Fold Changes of 15 selected DEGs using RNA-Seq and qRT-PCR.

**Additional file 2: Table S1**. Basic statistics of total raw and clean reads in RNA-Seq. **Table S2**. Mapping statistics of clean reads of RNA-Seq data to reference rice genome. **Table S3**. The primer sequences of genes validated using qRT-PCR assay.

## Data Availability

All raw reads of transcriptome data have been uploaded to the Bioproject PRJNA727740 (https://www.ncbi.nlm.nih.gov/sra/?term=PRJNA727740) and more detail information about the samples can be accessed via the SRA Run Selector link (https://www.ncbi.nlm.nih.gov/Traces/study/?acc=PRJNA727740&o=acc_s%3Aa). All relevant data are provided within the article and its supplementary information files.

## Abbreviations

EIA: Embryo side in air
CBW: Covered by water
DEGs: Differentially expressed genes
ABA: Abscisic acid
GA: Gibberellin
PCA: Principal component analysis
PCC: Pearson correlation coefficient
GO: Gene ontology
KEGG: Kyoto encyclopedia of genes and genomes
BP: Biological process
ROS: Reactive oxygen species
qRT-PCR: Quantitative real-time PCR

## References

[1] NODA A, HAYASHI J (1960). Studies on the coleorhiza of cereals.: VII. On the coleorhiza hair of rice-plant. Jpn J Crop Sci.

[2] MORITA O, EHARA H, GOTO M (1997). Anchoring function of coleorhiza hairs and seedling establishment of surface-sown forage grasses. Jpn J Grassland Sci.

[3] Bouchenak-Khelladi Y, Verboom GA, Savolainen V, Hodkinson TR (2010). Biogeography of the grasses (Poaceae): a phylogenetic approach to reveal evolutionary history in geographical space and geological time. Bot J Linn Soc.

[4] NODA A (1963). Studies on the coleorhiza of cereals. Memories Fac of Agr Kagawa Univ.

[5] Dowling P, Clements R, McWilliam J (1971). Establishment and survival of pasture species from seeds sown on the soil surface. Aust J Agric Res.

[6] Ma Z-h, Wang Y-b, Cheng H-t, G-c Z, Lyu W-y (2020). Biochemical composition distribution in different grain layers is associated with the edible quality of rice cultivars. Food Chem.

[7] Carrijo DR, Lundy ME, Linquist BA (2017). Rice yields and water use under alternate wetting and drying irrigation: a meta-analysis. Field Crops Res.

[8] Khush GS (1997). Origin, dispersal, cultivation and variation of rice. Plant Mol Biol.

[9] Sandhu N, Yadav S, Kumar Singh V, Kumar A (2021). Effective crop management and modern breeding strategies to ensure higher crop productivity under direct seeded Rice cultivation system: a review. Agronomy.

[10] Kumar V, Jat HS, Sharma PC, Balwinder-Singh GMK, Malik RK, Kamboj BR, Yadav AK, Ladha JK, Raman A (2018). Can productivity and profitability be enhanced in intensively managed cereal systems while reducing the environmental footprint of production? Assessing sustainable intensification options in the breadbasket of India. Agric Ecosyst Environ.

[11] Chakraborty D, Ladha JK, Rana DS, Jat ML, Gathala MK, Yadav S, Rao AN, Ramesha MS, Raman A (2017). A global analysis of alternative tillage and crop establishment practices for economically and environmentally efficient rice production. Sci Rep.

[12] Song T, Xu F, Yuan W, Chen M, Xu W (2019). Combining alternate wetting and drying irrigation with reduced phosphorus fertilizer application reduces water use and promotes phosphorus use efficiency without yield loss in rice plants. Agric Water Manage.

[13] Triant DA, Singh N, Mohanty B. Promoter architecture and transcriptional regulation of genes upregulated in germination and coleoptile elongation of diverse Rice genotypes tolerant to submergence. Front Genet. 2021;12:639654. 10.3389/fgene.2021.639654 PMC8008075 33796132

[14] Tang D, Guo H, Shi X, Wang Z (2019). Comparative transcriptome analysis of the gills from the Chinese mitten crab (Eriocheir japonica sinensis) exposed to the heavy metal Cadmium. Turk J Fish Aquat Sci.

[15] Chen M-X, Zhang Y, Fernie AR, Liu Y-G, Zhu F-Y. SWATH-MS-Based Proteomics: Strategies and Applications in Plants. Trends Biotechnol. 2021;39(5):433–7. 10.1016/j.tibtech.2020.09.002 33036785

[16] Wang G, Li H, Meng S, Yang J, Ye N, Zhang J (2020). Analysis of global methylome and gene expression during carbon reserve mobilization in stems under soil drying. Plant Physiol.

[17] Song T, Das D, Yang F, Chen M, Zhang J. Genome-wide transcriptome analysis of roots in two rice varieties in response to alternate wetting and drying irrigation. Crop J. 2020;8(4):586–601.

[18] Bain PA, Gregg AL, Kumar A (2016). De novo assembly and analysis of changes in the protein-coding transcriptome of the freshwater shrimp Paratya australiensis (Decapoda: Atyidae) in response to acid sulfate drainage water. BMC Genomics.

[19] Qiao Z, Pingault L, Zogli P, Langevin M, Rech N, Farmer A, Libault M (2017). A comparative genomic and transcriptomic analysis at the level of isolated root hair cells reveals new conserved root hair regulatory elements. Plant Mol Biol.

[20] Ranjan A, Pandey N, Lakhwani D, Dubey NK, Pathre UV, Sawant SV (2012). Comparative transcriptomic analysis of roots of contrasting Gossypium herbaceum genotypes revealing adaptation to drought. BMC Genomics.

[21] Yang C, Powell CA, Duan Y, Ancona V, Huang J, Zhang M (2020). Transcriptomic analysis reveals root metabolic alteration and induction of huanglongbing resistance by sulphonamide antibiotics in huanglongbing-affected citrus plants. Plant Pathol.

[22] Holloway T, Steinbrecher T, Perez M, Seville A, Stock D, Nakabayashi K, Leubner-Metzger G (2021). Coleorhiza-enforced seed dormancy: a novel mechanism to control germination in grasses. New Phytol.

[23] Nishimura M (1922). On the Germination and the Polyembryony of *Poa pratensis*, L. Shokubutsugaku Zasshi.

[24] Howarth W (1927). The seedling development of Festuca rubra L. var. tenuifolia Mihi, and its bearing on the morphology of the grass embryo. New Phytol.

[25] Norstog KJ: Responses of the oat coleorhiza to various treatments in culture. 1955.

[26] Zhang Y, Du H, Gui Y, Xu F, Liu J, Zhang J, Xu W (2020). Moderate water stress in rice induces rhizosheath formation associated with abscisic acid and auxin responses. J Exp Bot.

[27] Chandran AKN, Priatama RA, Kumar V, Xuan Y, Je BI, Kim CM, Jung K-H, Han C-D (2016). Genome-wide transcriptome analysis of expression in rice seedling roots in response to supplemental nitrogen. J Plant Physiol.

[28] Hu L, Xie Y, Fan S, Wang Z, Wang F, Zhang B, Li H, Song J, Kong L (2018). Comparative analysis of root transcriptome profiles between drought-tolerant and susceptible wheat genotypes in response to water stress. Plant Sci.

[29] Dalal M, Sahu S, Tiwari S, Rao AR, Gaikwad K (2018). Transcriptome analysis reveals interplay between hormones, ROS metabolism and cell wall biosynthesis for drought-induced root growth in wheat. Plant Physiol Biochem.

[30] Sun L, Di D-W, Li G, Li Y, Kronzucker HJ, Shi W (2020). Transcriptome analysis of rice (*Oryza sativa* L.) in response to ammonium resupply reveals the involvement of phytohormone signaling and the transcription factor OsJAZ9 in reprogramming of nitrogen uptake and metabolism. J Plant Physiol.

[31] Qian Q, Guo L, Smith SM, Li J (2016). Breeding high-yield superior quality hybrid super rice by rational design. Natl Sci Rev.

[32] Nguyen MX, Moon S, Jung K-H (2013). Genome-wide expression analysis of rice aquaporin genes and development of a functional gene network mediated by aquaporin expression in roots. Planta.

[33] Guo L, Wang ZY, Lin H, Cui WE, Chen J, Liu M, Chen ZL, Qu LJ, Gu H (2006). Expression and functional analysis of the rice plasma-membrane intrinsic protein gene family. Cell Res.

[34] Xu F, Wang K, Yuan W, Xu W, Liu S, Kronzucker HJ, Chen G, Miao R, Zhang M, Ding M (2019). Overexpression of rice aquaporin OsPIP1; 2 improves yield by enhancing mesophyll CO2 conductance and phloem sucrose transport. J Exp Bot.

[35] Sun JY, Liu XS, Khan IU, Wu XC, Yang ZM (2021). OsPIP2; 3 as an aquaporin contributes to rice resistance to water deficit but not to salt stress. Environ Exp Bot.

[36] Graether SP, Boddington KF (2014). Disorder and function: a review of the dehydrin protein family. Front Plant Sci.

[37] Vernoux T, Wilson RC, Seeley KA, Reichheld J-P, Muroy S, Brown S, Maughan SC, Cobbett CS, Van Montagu M, Inzé D (2000). The ROOT MERISTEMLESS1/CADMIUM SENSITIVE2 gene defines a glutathione-dependent pathway involved in initiation and maintenance of cell division during postembryonic root development. Plant Cell.

[38] Hasanuzzaman M, Bhuyan M, Parvin K, Bhuiyan TF, Anee TI, Nahar K, Hossen M, Zulfiqar F, Alam M, Fujita M (2020). Regulation of ROS metabolism in plants under environmental stress: a review of recent experimental evidence. Int J Mol Sci.

[39] Li Z, Gao Q, Liu Y, He C, Zhang X, Zhang J (2011). Overexpression of transcription factor ZmPTF1 improves low phosphate tolerance of maize by regulating carbon metabolism and root growth. Planta.

[40] Pompeiano A, Guglielminetti L (2016). Carbohydrate metabolism in germinating caryopses of Oryza sativa L. exposed to prolonged anoxia. J Plant Res.

[41] Pompeiano A, Fanucchi F, Guglielminetti L (2013). Amylolytic activity and carbohydrate levels in relation to coleoptile anoxic elongation in Oryza sativa genotypes. J Plant Res.

[42] Ali AS, Elozeiri AA. Metabolic processes during seed germination. Adv Seed Biol. 2017:141–66.

[43] Matilla AJ (2020). Auxin: hormonal signal required for seed development and dormancy. Plants.

[44] Wang T, Li C, Wu Z, Jia Y, Wang H, Sun S, Mao C, Wang X (2017). Abscisic acid regulates auxin homeostasis in rice root tips to promote root hair elongation. Front Plant Sci.

[45] Vishal B, Kumar PP (2018). Regulation of seed germination and abiotic stresses by gibberellins and abscisic acid. Front Plant Sci.

[46] Shu K, Liu X-d, Xie Q, He Z-h (2016). Two faces of one seed: hormonal regulation of dormancy and germination. Mol Plant.

[47] Laplaze L, Lucas M, Champion A (2015). Rhizobial root hair infection requires auxin signaling. Trends Plant Sci.

[48] Qin H, Huang R. Auxin controlled by ethylene steers root development. Int J Mol Sci. 2018;19(11):3656. 10.3390/ijms19113656 PMC6274790 30463285

[49] Zhang Y, Xu F, Ding Y, Du H, Zhang Q, Dang X, et al. Abscisic acid mediates barley rhizosheath formation under mild soil drying by promoting root hair growth and auxin response. Plant Cell Environ. 2021;44(6):1935–45. 10.1111/pce.14036 33629760

