# Supplementary material for: Comparative transcriptome analysis of coleorhiza development in japonica and Indica rice
Source: BMC Plant Biol. 2021 Nov 4;21:514. doi: 10.1186/s12870-021-03276-z (PMC8567703; doi:10.1186/s12870-021-03276-z)
Supplement: Supplementary file 1 — Additional file 1: Fig. S1. Correlation matrix heatmap visualizing the Pearson Correlation coefficient between RNA-Seq samples. Fig. S2. Volcano plot of differentially expressed genes (DEGs) for EIA vs. CBW treated samples. Fig. S3. Hierarchical clustering heatmap between RNA-Seq samples. Fig. S4. Overview of GO enrichment analysis of DEGs in japonica and indica from T1 and T2 stages. Fig. S5. Overview of top 10 KEGG pathways f DEGs in japonica and indica from T1 and T2 stages. Fig. S6. DEGs enriched in “glutathione metabolic process, response to oxidative stress, hydrogen peroxide catabolic process” BP categories of GO enrichments. Fig. S7. DEGs enriched in “carbon metabolism process” BP categories of GO enrichments. Fig. S8. DEGs enriched in “amino sugar and nucleotide sugar metabolism and fructose and mannose metabolism” BP categories of GO enrichments. Fig. S9. Comparison of the Fold Changes of 15 selected DEGs using RNA-Seq and qRT-PCR. [file 12870_2021_3276_MOESM1_ESM.docx]

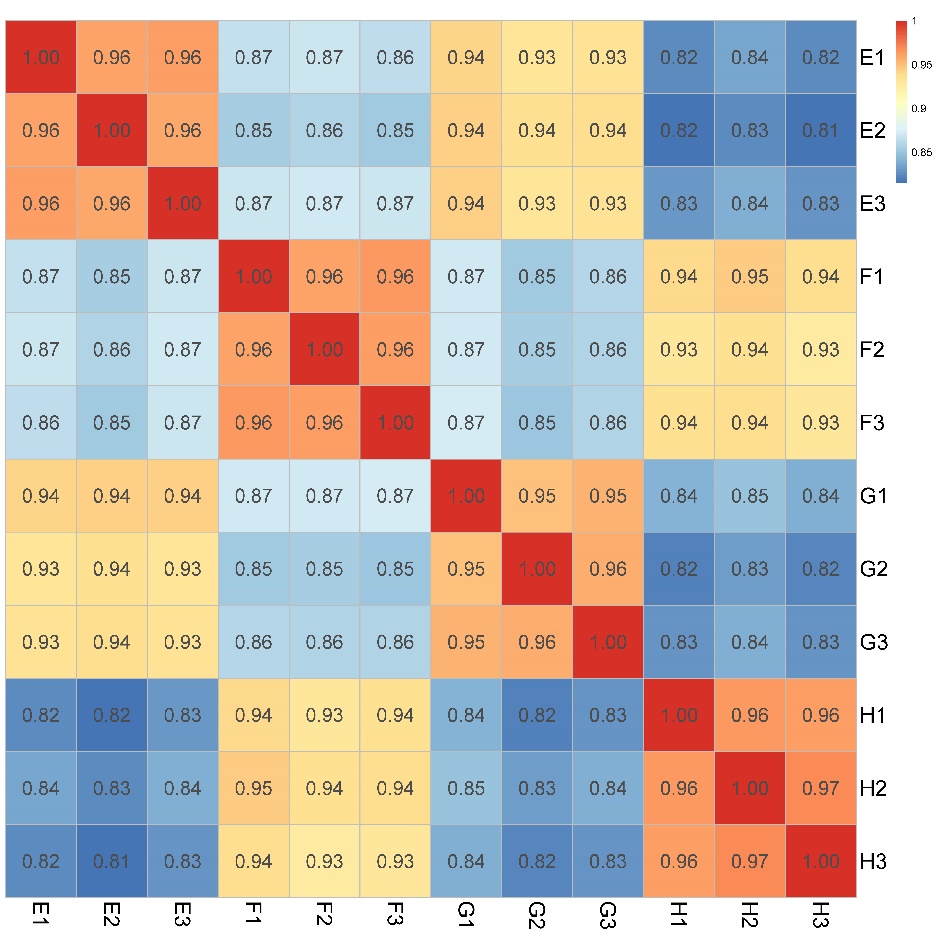

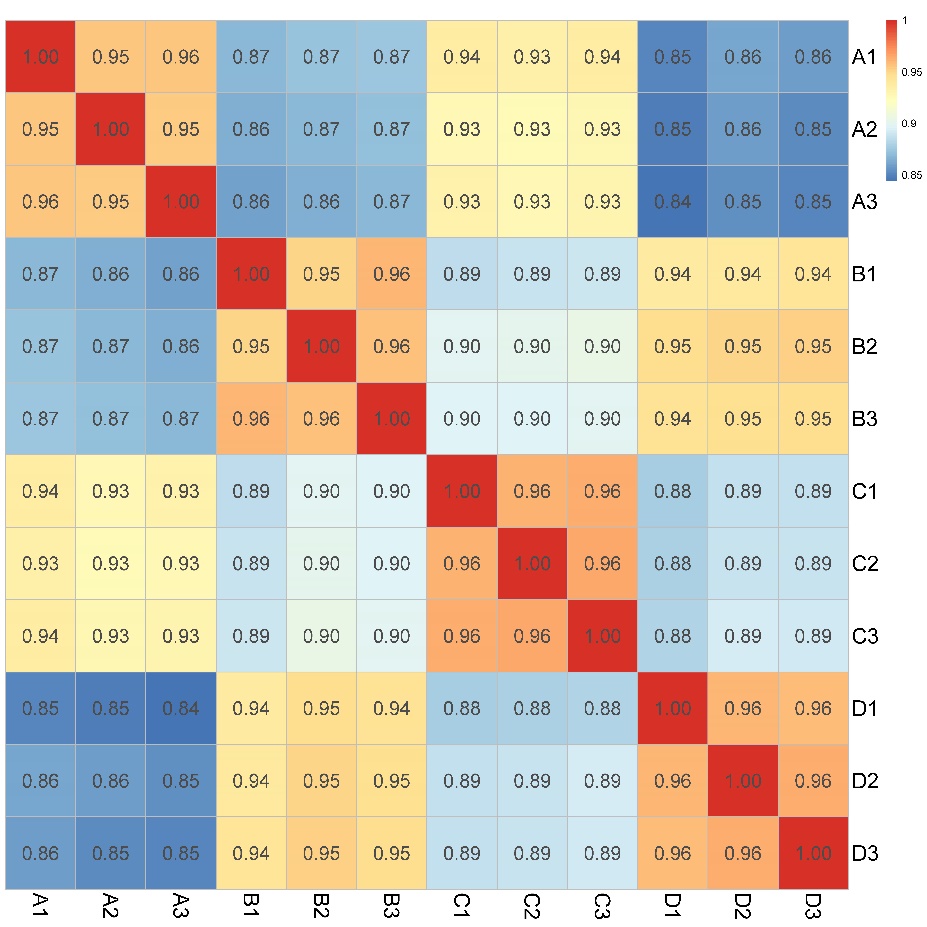
**Figure S1.** Correlation matrix heatmap visualizing the Pearson Correlation coefficient between RNA-Seq samples. (a), Pearson correlation assays among all samples in Nipponbare. (b), Pearson correlation assays among all samples in 9311. A and C represent CBW treated Nipponbare at T1 and T2, respectively; B and D represent EIA treated Nipponbare at T1 and T2, respectively; E and G represent CBW treated 9311 at T1 and T2, respectively; F and H represent EIA treated 9311 at T1 and T2, respectively; A1, A2 and A3 represents the three biological replicates of A.

**(b)**

**(a)**


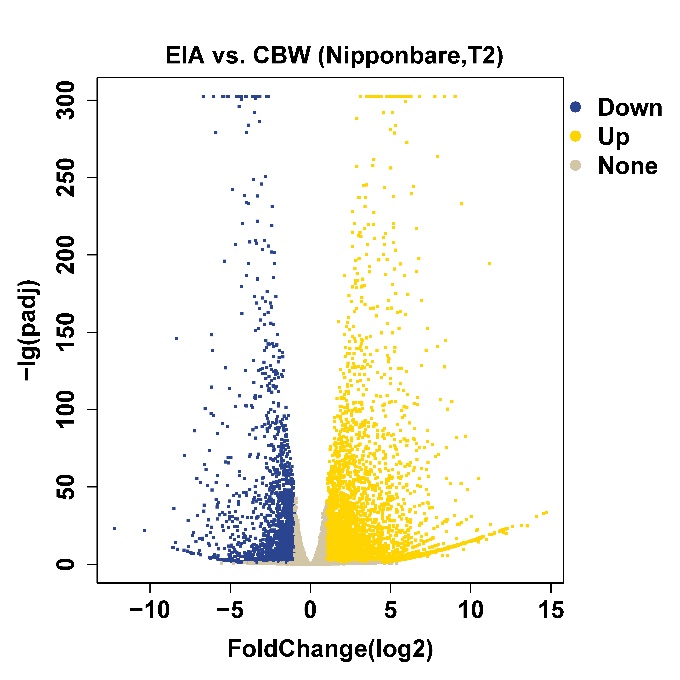
**Figure S2.** Volcano plot of differentially expressed genes (DEGs) for EIA vs CBW treated samples.
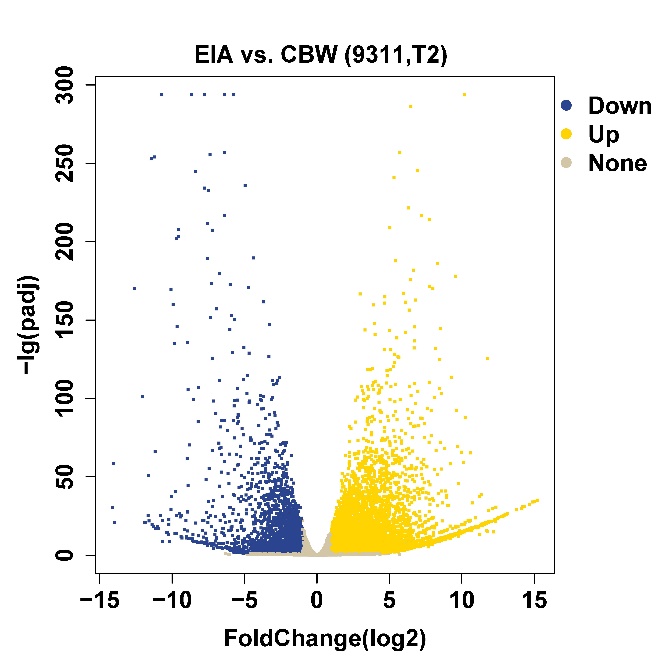

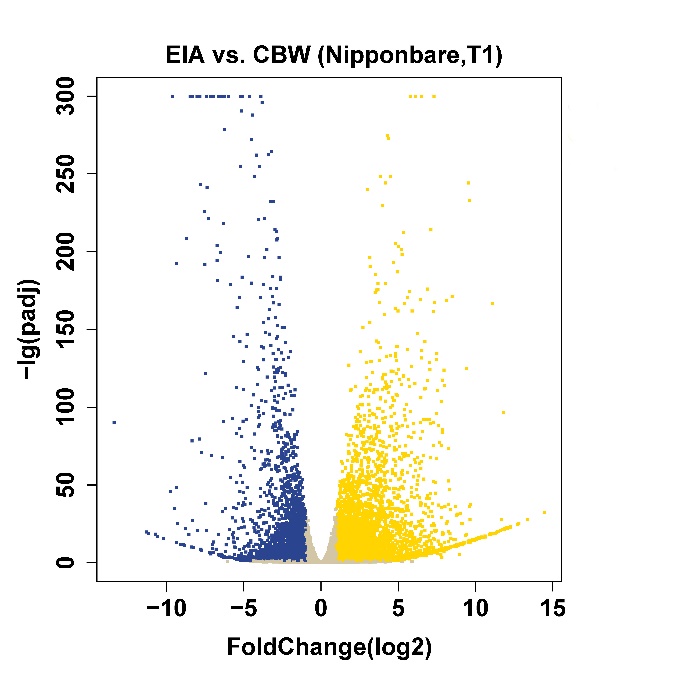

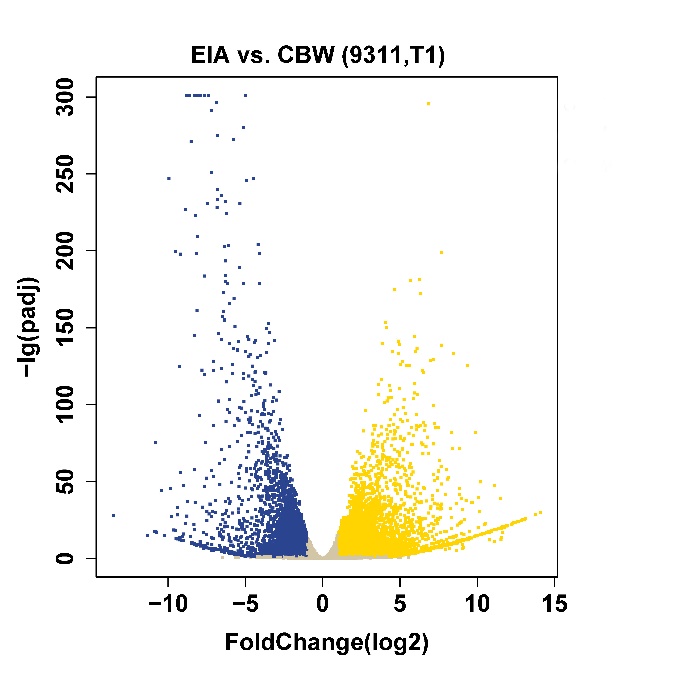


**(b)**

**(d)**

**(c)**

**(a)**

**
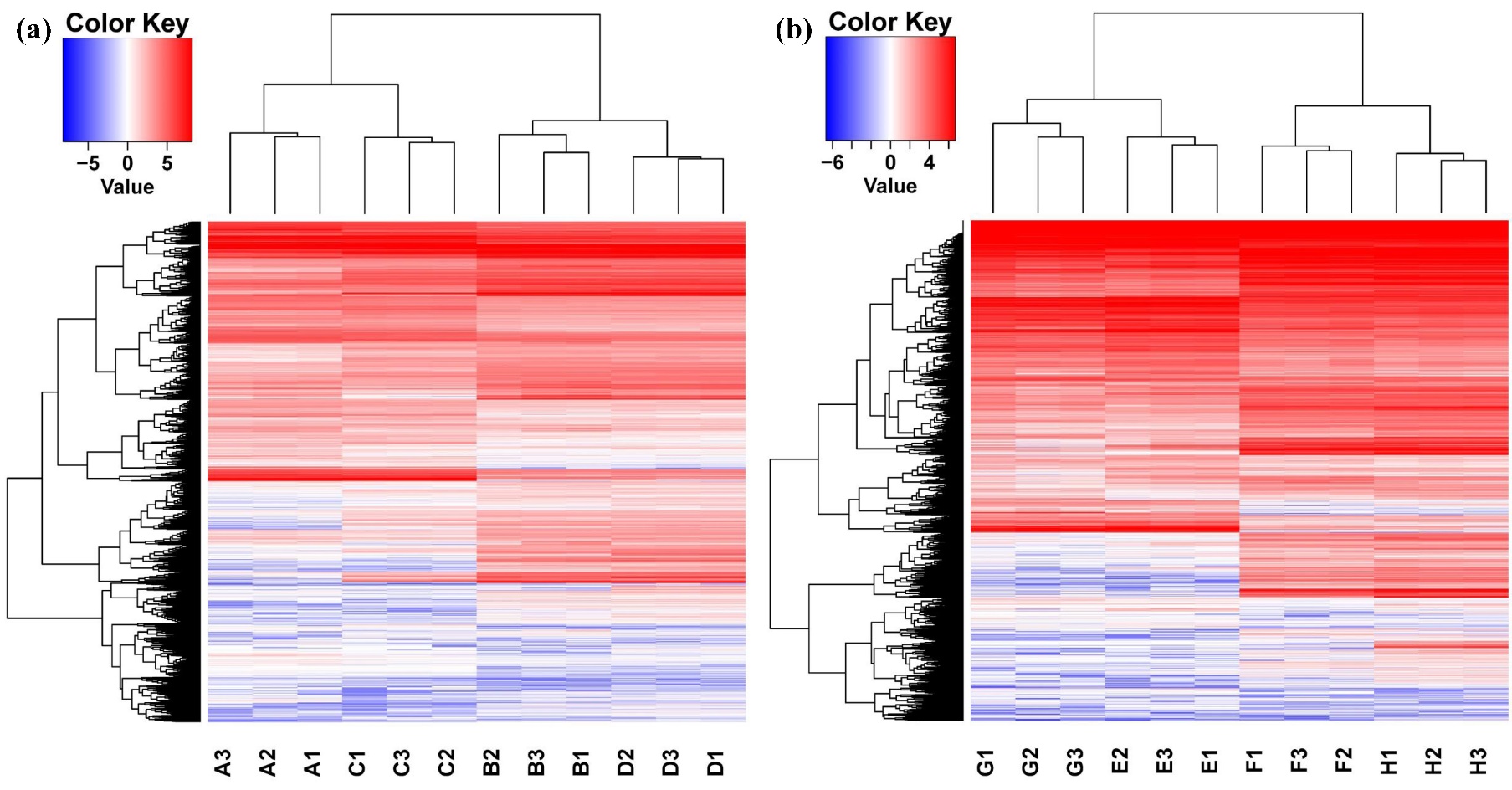
Figure S3.** Hierarchical clustering heatmap between RNA-Seq samples. (a), Hierarchical clustering heatmap in Nipponbare. (b), Hierarchical clustering heatmap in 9311. A and C represent CBW treated Nipponbare at T1 and T2, respectively; B and D represent EIA treated Nipponbare at T1 and T2, respectively; E and G represent CBW treated 9311 at T1 and T2, respectively; F and H represent EIA treated 9311 at T1 and T2, respectively; A1, A2 and A3 represents the three biological replicates of A.


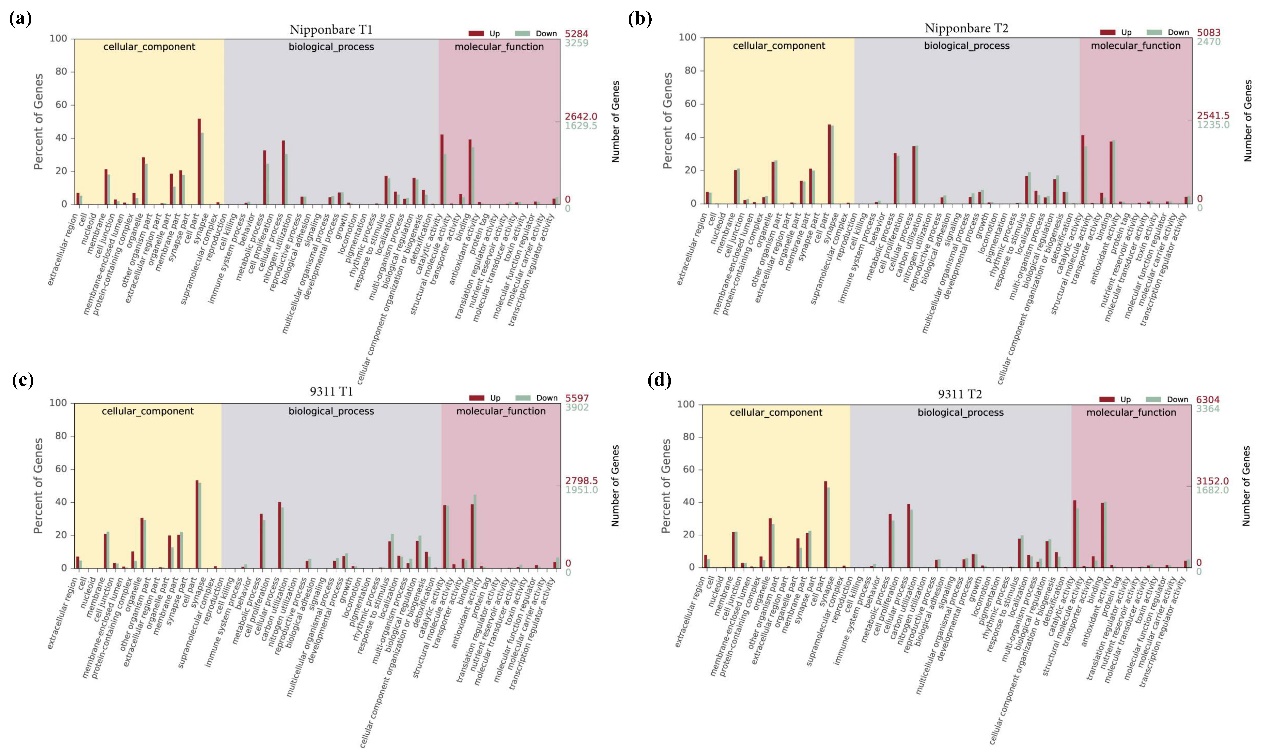
**Figure S4.** Overview of GO enrichment analysis of DEGs in *japonica* and *indica* from T1 and T2 stages.


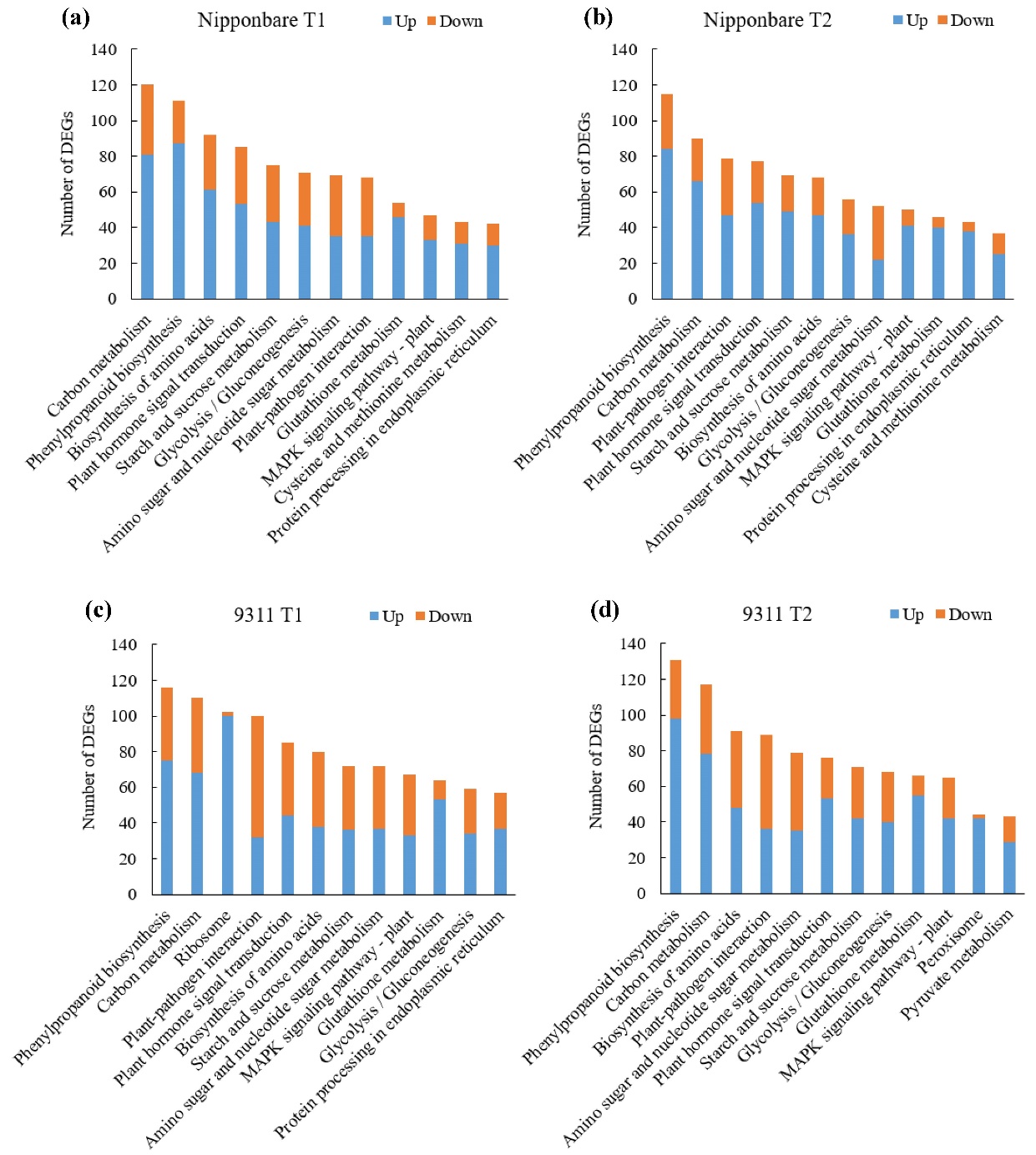
**Figure S5**. Overview of top 10 KEGG pathways f DEGs in *japonica* and *indica* from T1 and T2 stages.


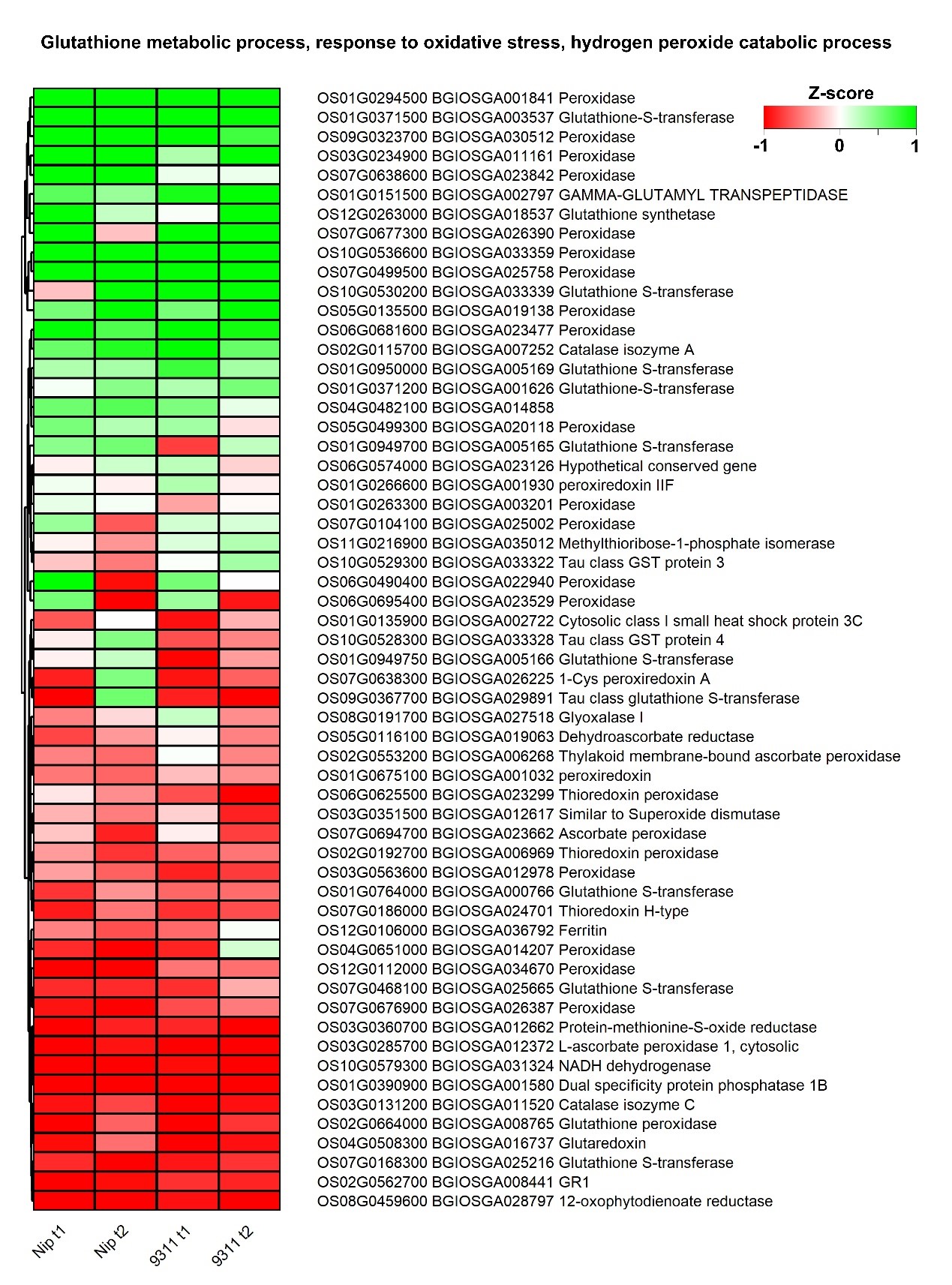
**Figure S6.** DEGs enriched in “glutathione metabolic process, response to oxidative stress, hydrogen peroxide catabolic process” BP categories of GO enrichments.


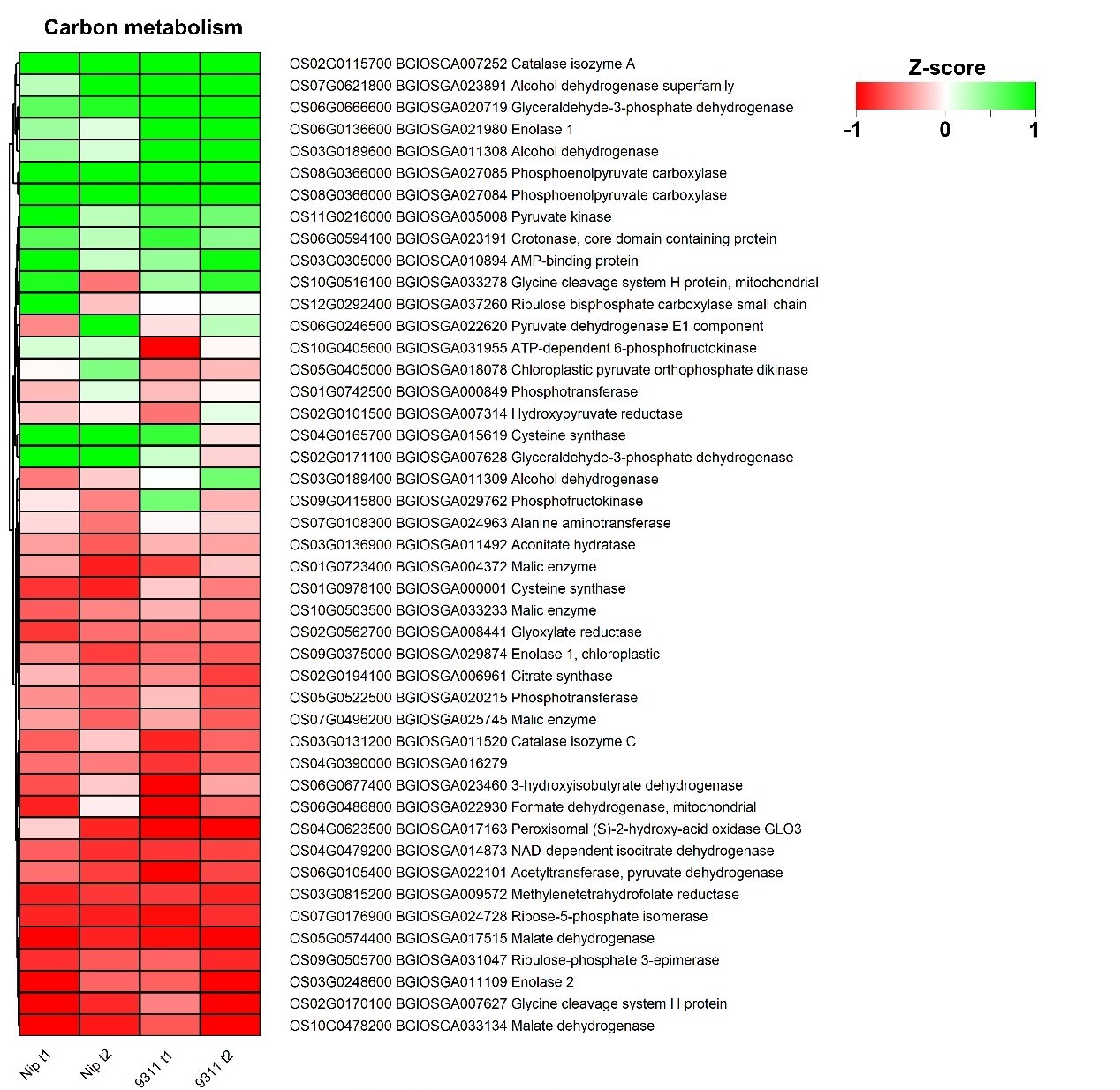
**Figure S7.** DEGs enriched in “carbon metabolism process” BP categories of GO enrichments.


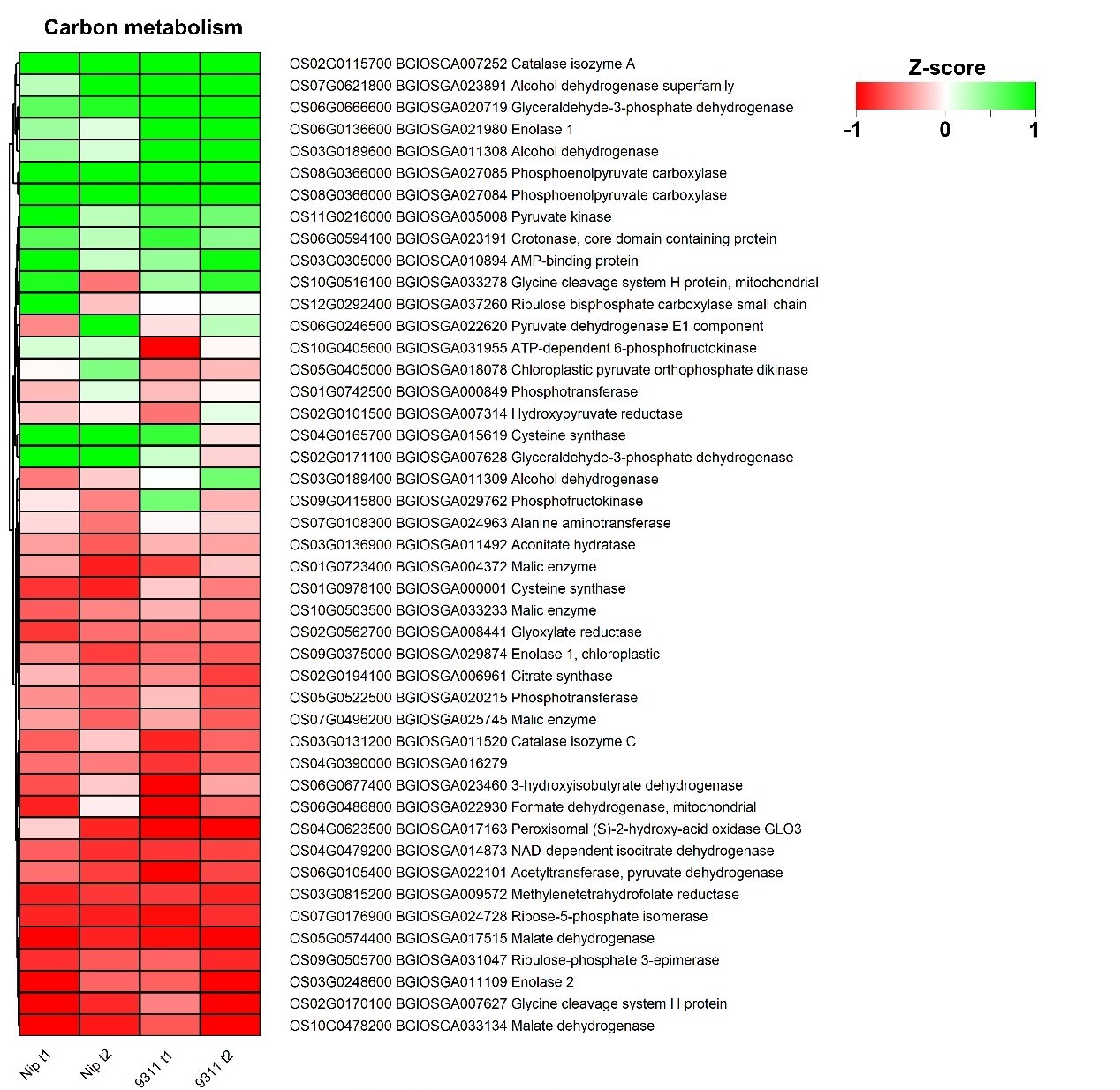
**Figure S8.** DEGs enriched in “amino sugar and nucleotide sugar metabolism and fructose and mannose metabolism” BP categories of GO enrichments.


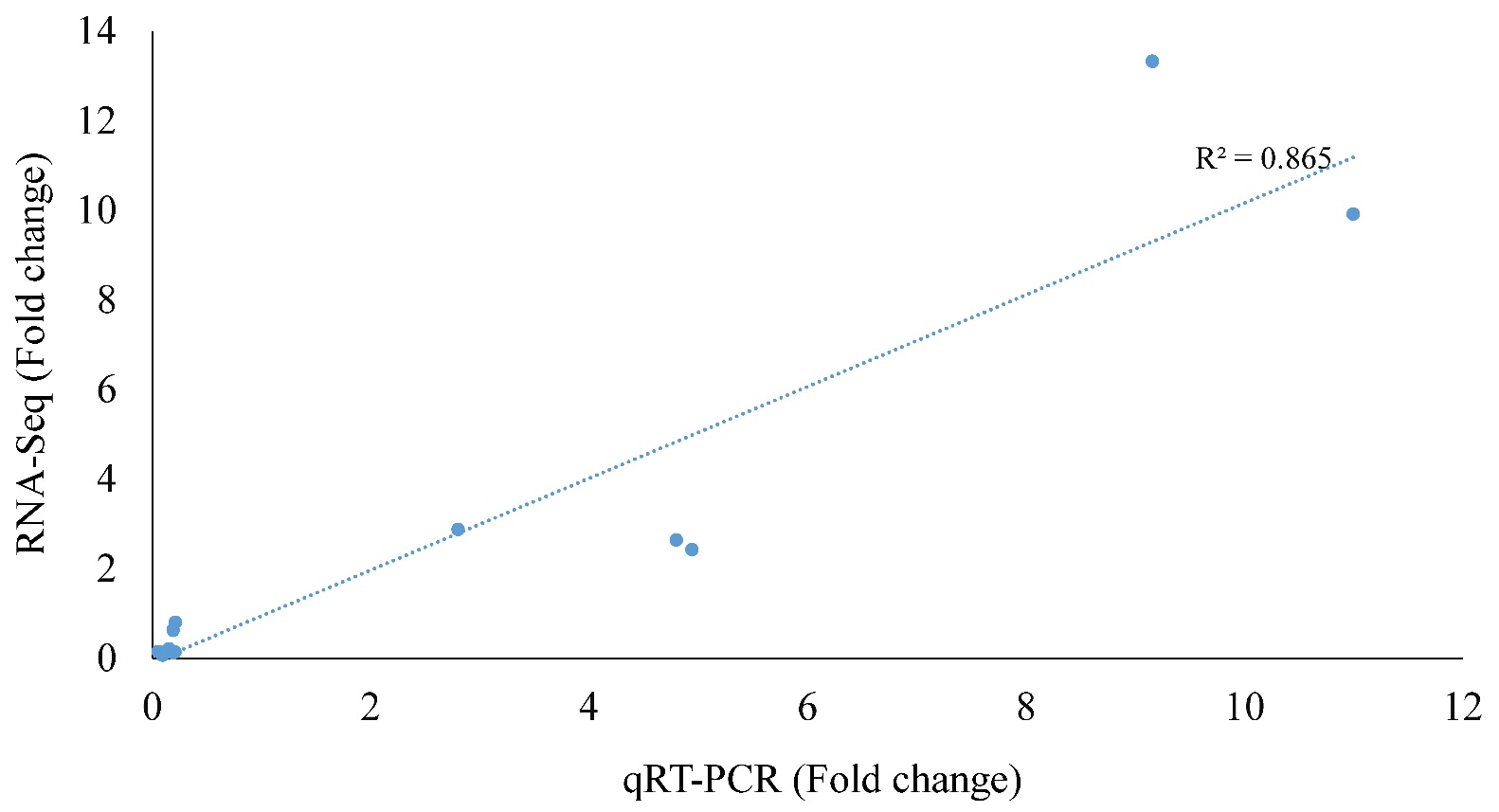


**Figure S9**. Comparison of the Fold Changes of 15 selected DEGs using RNA-Seq and qRT-PCR.
