# Supplementary material for: Comparative transcriptome analysis of coleorhiza development in japonica and Indica rice
Source: BMC Plant Biol. 2021 Nov 4;21:514. doi: 10.1186/s12870-021-03276-z (PMC8567703; doi:10.1186/s12870-021-03276-z)
Supplement: Supplementary file 2 — Additional file 2: Table S1. Basic statistics of total raw and clean reads in RNA-Seq. Table S2. Mapping statistics of clean reads of RNA-Seq data to reference rice genome. Table S3. The primer sequences of genes validated using qRT-PCR assay. [file 12870_2021_3276_MOESM2_ESM.docx]

**Table S1. Basic statistics of total raw and clean reads of transcriptome sequencing.**

| Sample | Raw Reads | Clean Reads | Raw Base(G) | Clean Base(G) | Q30(%) |
| --- | --- | --- | --- | --- | --- |
| NIP-CBW-T1-1 | 46290230 | 42926304 | 6.94 | 6.44 | 94.25 |
| NIP-CBW-T1-2 | 46993256 | 43332444 | 7.05 | 6.5 | 93.89 |
| NIP-CBW-T1-3 | 50847994 | 47669158 | 7.63 | 7.15 | 94.13 |
| NIP-EIA-T1-1 | 47413786 | 44213574 | 7.11 | 6.63 | 94.22 |
| NIP-EIA-T1-2 | 50817622 | 47316030 | 7.62 | 7.1 | 94.25 |
| NIP-EIA-T1-3 | 55766066 | 52314126 | 8.36 | 7.85 | 94.31 |
| NIP-CBW-T2-1 | 67398328 | 65500342 | 10.11 | 9.83 | 94.13 |
| NIP-CBW-T2-2 | 56311530 | 52048040 | 8.45 | 7.81 | 94.10 |
| NIP-CBW-T2-3 | 56370322 | 52565152 | 8.46 | 7.88 | 94.32 |
| NIP-EIA-T2-1 | 52171014 | 47861822 | 7.83 | 7.18 | 94.15 |
| NIP-EIA-T2-2 | 46828258 | 43416708 | 7.02 | 6.51 | 94.31 |
| NIP-EIA-T2-3 | 46973770 | 43197864 | 7.05 | 6.48 | 94.31 |
| 9311-CBW-T1-1 | 44913402 | 41408908 | 6.74 | 6.21 | 93.67 |
| 9311-CBW-T1-2 | 51491470 | 47926236 | 7.72 | 7.19 | 94.15 |
| 9311-CBW-T1-3 | 46548402 | 43712316 | 6.98 | 6.56 | 94.25 |
| 9311-EIA-T1-1 | 50978374 | 47746344 | 7.65 | 7.16 | 94.22 |
| 9311-EIA-T1-2 | 40870452 | 37441114 | 6.13 | 5.62 | 93.83 |
| 9311-EIA-T1-3 | 47627216 | 44539830 | 7.14 | 6.68 | 94.46 |
| 9311-CBW-T2-1 | 44369474 | 41953852 | 6.66 | 6.29 | 94.10 |
| 9311-CBW-T2-2 | 57719290 | 53512446 | 8.66 | 8.03 | 94.12 |
| 9311-CBW-T2-3 | 42269062 | 39098972 | 6.34 | 5.86 | 94.34 |
| 9311-EIA-T2-1 | 44346028 | 41581950 | 6.65 | 6.24 | 94.12 |
| 9311-EIA-T2-2 | 68805976 | 64208798 | 10.32 | 9.63 | 94.25 |
| 9311-EIA-T2-3 | 42360058 | 39624890 | 6.35 | 5.94 | 94.06 |

Nip: Nipponbare, EIA: embryo side in air ,CBW: covered with water.

**Table S2. Mapping statistics of clean reads of RNA sequencing data to reference rice genome.**

| Sample | Mapped Reads | Mapping Rate | UnMapped Reads | MultiMap Reads | MultiMap Rate |
| --- | --- | --- | --- | --- | --- |
| NIP-CBW-T1-1 | 41957063 | 0.9774 | 969241 | 2152451 | 0.0501 |
| NIP-CBW-T1-2 | 42314052 | 0.9765 | 1018392 | 2145997 | 0.0495 |
| NIP-CBW-T1-3 | 46602526 | 0.9776 | 1066632 | 2315886 | 0.0486 |
| NIP-EIA-T1-1 | 43160686 | 0.9762 | 1052888 | 1911381 | 0.0432 |
| NIP-EIA-T1-2 | 46087758 | 0.974 | 1228272 | 2152464 | 0.0455 |
| NIP-EIA-T1-3 | 51215183 | 0.979 | 1098943 | 2477102 | 0.0474 |
| NIP-CBW-T2-1 | 64002766 | 0.9771 | 1497576 | 3308713 | 0.0505 |
| NIP-CBW-T2-2 | 50852214 | 0.977 | 1195826 | 2473637 | 0.0475 |
| NIP-CBW-T2-3 | 51310103 | 0.9761 | 1255049 | 2574785 | 0.049 |
| NIP-EIA-T2-1 | 42380321 | 0.9761 | 1036387 | 2067521 | 0.0476 |
| NIP-EIA-T2-2 | 46686476 | 0.9754 | 1175346 | 2201571 | 0.046 |
| NIP-EIA-T2-3 | 42183398 | 0.9765 | 1014466 | 1940267 | 0.0449 |
| 9311-CBW-T1-1 | 39500653 | 0.9539 | 1908255 | 2858533 | 0.069 |
| 9311-CBW-T1-2 | 45815979 | 0.956 | 2110257 | 3472091 | 0.0724 |
| 9311-CBW-T1-3 | 41713057 | 0.9543 | 1999259 | 2837213 | 0.0649 |
| 9311-EIA-T1-1 | 45713791 | 0.9574 | 2032553 | 2828531 | 0.0592 |
| 9311-EIA-T1-2 | 35772609 | 0.9554 | 1668505 | 1991110 | 0.0532 |
| 9311-EIA-T1-3 | 42764548 | 0.9601 | 1775282 | 2477109 | 0.0556 |
| 9311-CBW-T2-1 | 39949159 | 0.9522 | 2004693 | 3046566 | 0.0726 |
| 9311-CBW-T2-2 | 51003320 | 0.9531 | 2509126 | 3899596 | 0.0729 |
| 9311-CBW-T2-3 | 37134769 | 0.9498 | 1964203 | 4051133 | 0.1036 |
| 9311-EIA-T2-1 | 39785106 | 0.9568 | 1796844 | 2596798 | 0.0625 |
| 9311-EIA-T2-2 | 61356565 | 0.9556 | 2852233 | 3742139 | 0.0583 |
| 9311-EIA-T2-3 | 37910302 | 0.9567 | 1714588 | 2452166 | 0.0619 |

**Table S3 –Primers used for qRT-PCR.**

| **Cultivar** | **Gene ID** | **Primer sequence (5'–3')** | **RNA-Seq (fold change)** | **qRT-PCR (fold change)** |
| --- | --- | --- | --- | --- |
| Nipponbare-T1 | *OS01G0764800* | \| CAGGAGGCAGATGCTCTACA \| \| --- \| \| CGTCTTCGTCTCCGACTTGA \| | 0.18 | 0.15 |
| Nipponbare-T1 | *OS07G0154100* | \| TCGGGAGGTACGACTTCCAT \| \| --- \| \| GGCTTGGACACGACATTGTA \| | 11.00 | 9.91 |
| Nipponbare-T1 | *OS03G0297600* | \| GGAGGTGCCTATGGAGGTG \| \| --- \| \| GGACGAACCGCTTGTACG \| | 0.068 | 0.14 |
| Nipponbare-T1 | *OS05G0473000* | \| CACCGCCTCTCCAACTACC \| \| --- \| \| TCCACCACGTAGGACTCCA \| | 0.15 | 0.21 |
| Nipponbare-T2 | *OS03G0131200* | \| GGCAAGACCGGTTCATCAAG \| \| --- \| \| AGCCTGAGACCAGTAGGAGA \| | 4.94 | 2.41 |
| Nipponbare-T2 | *OS05G0572700* | \| GAGTTGGAACGAGTGGAAGC \| \| --- \| \| ACGGCTTCAGGTAGTAGTCG \| | 0.21 | 0.14 |
| Nipponbare-T2 | *OS01G0273800* | \| GGAATCTACGGCAGCTACGA \| \| --- \| \| CGAGCCATTGTTGTGGGTAG \| | 4.80 | 2.63 |
| Nipponbare-T2 | *OS01G0764800* | \| CAGGAGGCAGATGCTCTACA \| \| --- \| \| CGTCTTCGTCTCCGACTTGA \| | 0.20 | 0.12 |
| 9311-T1 | *BGIOSGA032502* | \| ACACCCTCACGCTCACATAA \| \| --- \| \| CCTAACAGTGGGAAGCAGGA \| | 2.80 | 2.87 |
| 9311-T1 | *BGIOSGA017524* | \| AGGCGACTACTACCTGAAGC \| \| --- \| \| TCAGGAACTCGTCCTTGTCC \| | 0.10 | 0.067 |
| 9311-T1 | *BGIOSGA004510* | \| CAGGAGGCAGATGCTCTACA \| \| --- \| \| CGTCTTCGTCTCCGACTTGA \| | 0.20 | 0.62 |
| 9311-T1 | *BGIOSGA006838* | \| TGATGGTGACAAGAGCTCCA \| \| --- \| \| TGTGCCTCTTCTTCCTCTGG \| | 9.16 | 13.32 |
| 9311-T2 | *BGIOSGA010919* | \| GGAGGTGCCTATGGAGGTG \| \| --- \| \| GGACGAACCGCTTGTACG \| | 0.066 | 0.14 |
| 9311-T2 | *BGIOSGA004510* | \| CAGGAGGCAGATGCTCTACA \| \| --- \| \| CGTCTTCGTCTCCGACTTGA \| | 0.21 | 0.80 |
| 9311-T2 | *BGIOSGA017524* | \| AGGCGACTACTACCTGAAGC \| \| --- \| \| TCAGGAACTCGTCCTTGTCC \| | 0.056 | 0.15 |
| Nipponbare  and 9311 | *Os03g0718100/*  *BGIOSGA013463*  *(Actin1)* | GCATCTCTCAGCACATTCCA  CTGGTACCCTCATCAGGCAT | - | - |
